# Framework synthesis to inform the ideation and design of a paper‐based health information system (PHISICC)

**DOI:** 10.1002/hpm.3487

**Published:** 2022-04-23

**Authors:** Meike‐Kathrin Zuske, Christian Auer, Sandy Oliver, John Eyers, Xavier Bosch‐Capblanch

**Affiliations:** ^1^ Swiss Tropical and Public Health Institute Allschwil Switzerland; ^2^ University of Basel Basel Switzerland; ^3^ University College London EPPI‐Centre Social Research Institute London UK; ^4^ University of Johannesburg Africa Centre for Evidence Faculty of Humanities Johannesburg South Africa; ^5^ Independent Consultant & Senior Research Fellow, 3ie, c/o LIDC London UK

**Keywords:** decision‐making, framework, health information system, primary health care, systematic review

## Abstract

**Background:**

Health information systems (HIS) are meant to support decision‐making at all levels of the system, including frontline health workers. In field studies in Côte d’Ivoire, Mozambique and Nigeria, we observed health workers' interactions with the HIS and identified twelve decision‐making components of HIS. The objective of this framework synthesis is to portray these components in HIS research, in order to inform the ideation of a paper‐based HIS intervention (PHISICC).

**Methods:**

We searched studies in the Cochrane Central Register of Controlled Trials (CENTRAL), Embase, Epistemonikos, Medline, in‐Process on the Ovid platform, OpenGrey, PDQ  Evidence (“pretty darnd quick” Evidence), the World Health Organization (WHO) Global Health Library and included studies focussing on HIS interventions, data quality, information support tools and data use for decision‐making in the context of the governmental health care sector. We assessed the methodological quality of studies using the Critical Appraisal Skills Programme tool. We synthesised the findings based on the decision‐making components of HIS and thematic areas.

**Results:**

The search identified 6784 studies; 50 were included. Most of the 50 studies had quality concerns. All studies included at least one of the decision‐making components: the most prominent were the technical aspects of ‘recording’ and ‘reporting’. Data use for decision‐making was much less represented.

**Conclusion:**

HIS research focuses on the more technical aspects of HIS. Further research on HIS, given the strong push towards HIS digitalisation, should consider putting at the centre the human experience of decision‐making and data use, in order to make HIS relevant for quality of care.

## Highlights

- Across the health system, Health Information Systems (HIS) are meant to support data management and use, which are key activities of health care delivery.
- Informed by existing frameworks and by fieldwork in three African countries, we identified twelve decision‐making components of HIS.
- In this framework synthesis, we found that the most common decision‐making components in the HIS literature were those related to the technical aspects of data ‘recording’ and ‘reporting’; data use for decision‐making was much less represented.
- For HIS instruments to facilitate improved health care delivery, clinical and public health decision‐making components of HIS should be put at the centre for HIS research, ideation and implementation.

## 1 INTRODUCTION

The health and wellbeing of individuals and populations is directly affected by decisions made by policy makers and planners, managers, health care providers and by the population itself.^1^ Health Information Systems (HIS) are meant to inform decision‐making across all levels of the healthcare system.^1^ Information within health services is typically produced through Routine Health Information Systems (RHIS) or Health Management Information Systems (HMIS). Most of the HIS definitions and approaches are circumscribed to RHIS or HMIS and have a technical focus on the mechanisms and tools by which data and information are produced, supported and transformed.^2^, ^3^ However, decisions are also made based on other information sources, such as research, evaluation reports or even less formal sources, such as colloquial, personal or experiential evidence.^4^, ^5^, ^6^

In the frame of a multi‐country, transdisciplinary research project (Paper‐based Health Information System in Comprehensive Care, PHISICC^7^, ^8^) we recently carried out an effectiveness systematic review on the effects of interventions to improve HIS. This evidence was complemented with the characterisation of the HIS in the three countries where PHISICC took place (Côte d’Ivoire, Mozambique and Nigeria), which together with the effectiveness systematic review and the framework synthesis we present here informed the ideation and design of the intervention. The PHISICC intervention consisted of a suite of paper‐based tools for antenatal care, delivery, postnatal care, vaccination, sick child, general consultation, Human Immunodeficiency Virus (HIV) and tuberculosis. The tools shared a standard visual language with decision‐making hints, as a clinical decision support tool. The intervention was tested in three randomised controlled trials.

The rationale for a framework synthesis was twofold; one, an effectiveness review assessing the effects of interventions targeting HIS on the quality and use of data and on health‐related outcomes in primary health care (PHC) in low and middle‐income countries.^9^, ^10^ This systematic review was not primarily conceived to understand how HIS interventions work or how users interact with them, and had limited findings on those issues. Two, preliminary research carried out in the three PHISICC countries, using stakeholders' consultations as well as a mix of public health and Human Centred Design approaches, emphasised the need to refocus HIS research, from the more technical issues related to data management to the actual use of data for decision‐making by frontline health workers. The field work allowed us to identify a series of components of frontline health workers' decision‐making to guide our thinking (see Table 1). The list was produced following an inductive approach based on the nature of decision‐making, decision‐making domains and levels of decision‐making identified in the field.

**TABLE 1:** The 12 decision‐making components of HIS

	Component	Description	Example of information	Example of process
(A) Information for immediate decision‐making				
A.1	Assessing a situation	This component entails the interpretation of recorded or reported data on observed facts in order to assess a given situation. HIS are meant to facilitate this component by capturing the critical information in each setting (i.e. clinical, managerial or policy).	Clinical signs; vaccination status	Making diagnoses
A.2	Best course of action	The HIS is used to document decisions made in clinical or managerial context. This information is linked to A.1, through instructions and guidelines (B.6 and B.7).	Medication prescribed; vaccine type and number	Prescribing treatments
A.3	Outcomes	This component gives consideration to the outcomes or results of the decisions, which depend on the best course of action but also on other issues, such as adherence to recommendations or contextual issues that determine adherence to the best course of action.	Referral note for pregnancy at risk	Considering chances of successful delivery under certain conditions
A.4	Follow‐up	Some events require follow‐up and iterative processes to continuously ‘assess’ situations and choose or adapt interventions (e.g. chronic conditions, humanitarian emergencies). HIS should facilitate the recording and retrieval of longitudinal data for these situations.	Dates for complementary exams	Scheduling next visits
**(B) Information to document health care events (deferred decision‐making)**				
B.5	Transforming information	Very often data need to be transformed (i.e. analysed, converted, represented) in order to be used. HIS incorporate analytical and dissemination components to facilitate data use.	Aggregation of vaccinations by vaccine and month	Analyses
B.6	Reporting information	Information is relevant to several tiers of the health system: From individual patient care up to international health strategic decision‐making. HIS should have clear and robust reporting mechanisms.	Monthly reports of health facility activities	Sending information out to higher levels of the system
**(C) Information to maintain or improve the quality of health care**				
C.7	Technical guidance	HIS are also an opportunity to provide technical guidance to users and decision‐making. HIS can incorporate hints or reference materials to assist in the decision‐making processes.	Reference normal values of laboratory test or clinical features	Checking reference values while providing care
C.8	Acquiring and using knowledge	‘Knowledge’ includes all sorts of additional information that is often required to assess the situation, choose a course of action and accomplish sound decision‐making (e.g. contextual information, standing orders, trends). HIS should encompass routine information from health services but also demographic information and knowledge from research, to mention only two examples.	Clinical guidelines	Consultation of guidelines in the context of health care
C.9	Auditing health care	Evidence suggests that clinical audits may improve quality of care. HIS are shaped in a way that facilitates auditing.	Checklist to assess consistency of patients' records	Review of patients' records on supervision
C.10	Communication	Communication refers to the act of conveying a piece of information to someone else in an accurate and timely fashion, mostly because this communication is essential in order to produce reliable assessments and encourage adherence to best courses of action. In one way or another, communication takes place, in all health care events; between health workers and patients or with community members. HIS support communication by providing clear information items that can be understood by patients, providers, managers and policy makers, depending on the setting.	Side effects of treatments; alternative treatments	Discussions while consulting
**(D) Information system use**				
D.11	Data audit	Auditing and the other components of decision‐making require an idea on the accuracy and precision of data. HIS should incorporate routine mechanisms that allow checking the quality of data.	Events reported in monthly reports versus events recounted on the spot	Review data quality criteria on supervision
D.12	Help with the health information system	Finally, the HIS can be built with validation rules, constraints and other artefacts that can ease using the paper and digital data support tools.	Instructions on how to complete forms	While in clinical care or handling data.

The objectives of this framework synthesis were (i) to map the frontline health workers' decision‐making components in the research literature; (ii) to explore the links between information and its use; and (iii) to describe barriers and opportunities for information use for decision‐making within HIS. This framework synthesis contributed to inform the ideation of an innovative paper‐based HIS (PHISICC).

## 2 MATERIALS AND METHODS

We carried out a systematic review of the literature to produce a framework synthesis,^11^ and reported it following the Preferred Reporting Items for Systematic Reviews and Meta‐Analyses (PRISMA) reporting items (see Supplementary file 1). We implemented a search strategy to the following sources in April 2016: CENTRAL (The Cochrane Central Register of Controlled Trials), Embase, Epistemonikos, Medline, in‐Process on the Ovid platform, OpenGrey, PDQ Evidence (“pretty darn quick” Evidence), the World Health Organization (WHO) Global Health Library and a reference list of the chair of our technical advisory group. The search included terms related to HIS and decision‐making and was not adapted or limited to the specific decision‐making components in Table 1, because we precisely aimed at identifying to which extent we could map these components ‘from the field’ in the HIS literature. The complete search strategy for the observational studies, including qualitative research and case studies, can be found in the Supplementary file 2.

The studies' inclusion criteria were: observational, qualitative and case studies, addressing decision‐making capacity by PHC frontline health workers (i.e. nurses, clinicians, midwives as well as community health workers) or managers at district or at higher levels of the system. Studies related to patients' decision‐making were included if an information tool, device or subsystem was used by the health workers as well. We included studies containing information on perceptions, experiences and lessons learnt relevant for decision‐making and in relation to the supportive function of HIS in the PHC system. In cases where the main focus of a study was not on decision‐making components or the supportive components of data, tools or applications, the study was still included, if: (a) the characteristics of the problems described in the study were related to decision‐making or the use of data, tools or applications; (b) the findings, discussion or conclusions of the study were related to relevant components, problems or solutions for decision‐making processes or for the use of data, tools or applications. Studies had to refer to HIS interventions, data quality or information tools and provide any evidence in the results or discussion on barriers or facilitators of decision‐making.

We considered studies in the context of the governmental, public PHC system or where the health care context was mixed or not clearly identifiable. Studies conducted in private health care facilities or hospitals were excluded. We included studies carried out in any country and in any language.

First, we screened titles and abstracts for relevance. Studies that could not be clearly ruled out as irrelevant to our aims were kept and the full texts were retrieved to apply the inclusion criteria. Articles were screened only once by any of the reviewers, being inclusive in case of doubt. At inclusion/exclusion stage, some articles were double scrutinised by a second author (either Xavier Bosch‐Capblanch (XBC) or Christian Auer (CAU)) either because of doubts by the first reviewer Meike‐Kathrin Zuske (MZU) or arbitrarily as data were shared among the reviewers team. At least half of the articles would have been inspected by at least two reviewers. Data from the included studies were entered into a data extraction form specifically designed for this review to record the characteristics of studies, the mapping of decisions‐making components and statements related to the main findings. The form was also used for the quality assessment. The methodological quality of the studies was assessed by a single reviewer supervised by the corresponding author, using the Critical Appraisal Skills Programme (CASP) tool.^12^ Since different study designs were included, we selected the CASP criteria in order to have a common set of items across all observational study designs. Eleven questions were used and a quality score for each study was calculated by adding up positive answers. Studies were classified as having no concern (11 items passed), minor concerns (9 or 10 items), moderate‐minor concerns (7 or 8 items), moderate concerns (5–6 items), moderate‐serious concerns (2–4 items), or serious concerns (0 or 1 items passed).

For the first objective (mapping of decision‐making components), we considered all included studies. When a component was found within a study, this was marked as a ‘Yes’ in the correspondent data sheet; if a component was clearly not mentioned in a study, then we marked it as a ‘No’; if unclear, we recorded it as such. We used frequency analysis to populate the decision‐making components, by summing the positive hits for each component.

For the objectives two and three, we selected a subset of included studies with higher quality scores, in order to avoid unnecessary bias which would affect the synthesis of findings. After an initial coding of extracted findings in the form of statements, these were charted thematically^11^ and grouped into barriers, opportunities and strategies for the use of information.

## 3 RESULTS

**FIGURE 1:**
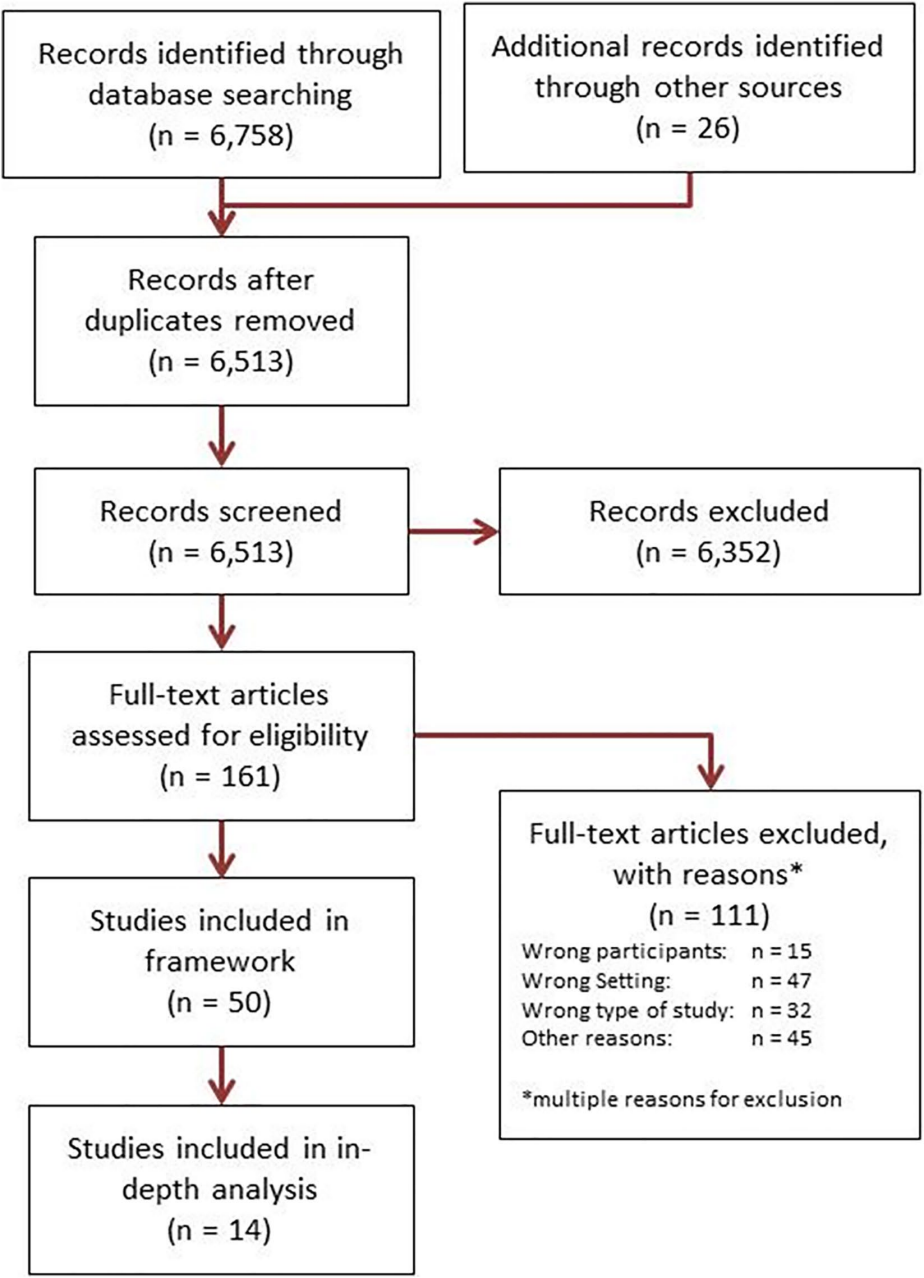
Flow of studies

The search strategy produced 6784 records. After removing 271 duplicates, the remaining 6513 records were screened for relevance by titles and abstracts and 6352 were considered irrelevant. We retrieved and screened the full reports of the remaining relevant 161 studies. Exclusions related to the setting of the study (not PHC, 47 studies), the topic not focussing on decision‐making (32 studies), wrong study design (32 studies) and other types of participants (15 studies). Fifty studies were included into the framework synthesis, objective 1, and for the in‐depth analysis (objectives 2 and 3), we included all 14 high‐quality studies (see Figure 1). See Supplementary file 3 for the list of included and excluded studies.

### 3.1 Characteristics of included studies

The characteristics of included studies are summarised in Table 2 and Supplementary file 4. The publication years of the included 50 studies ranged from 1994 to 2015 (median 2009). Study designs were: 31 cross‐sectional; eight mixed‐methods; eight qualitative; two case control and one cohort study. Of the included studies, 29 were conducted in PHC facilities, seven at several health care levels; and two at the district level. In 12 studies, the setting was not clearly identifiable.

**TABLE 2:** Characteristics of included studies

Characteristics of included studies	N
Type of study	
Cohort	1
Cross‐sectional	31
Case‐control	2
Qualitative	8
Mixed‐method	8
**Setting**	
PHC	29
District	2
Mixed	7
Unclear	12
**Country income level classification**	
High income country HIC	7
Upper‐middle income country UMIC	24
Lower‐middle income country LMIC	9
Low income country LIC	10
**Participants**	
Health care providers	31
Manager	2
Patients	5
Combination	12
**Health care area**	
Maternal and child health	16
General practice	10
Infectious diseases	3
Health information	3
Cancer care or prevention	2
Diabetes	2
Family health	2
Hypertension	2
Combination	2
Health financing	1
HIV/Aids	1
Malaria	1
Reproductive health	1
Tuberculosis	1
Unclear	3
**Study object**	
Data tool	36
Data quality	7
Combination	7

The majority of the studies were located in middle‐income countries (24 in upper‐middle and nine in lower‐middle income countries). Ten studies were conducted in low‐income countries (as per 2017): Benin, Ethiopia, Mali, Rwanda, Tanzania and Uganda. Seven of the included studies were conducted in high‐income countries: Israel, Singapore, Kuwait, Bahrain and in the United States of America (Hawaii).

Participants in 31 studies were health care providers. In five studies, patients were included because the use of a HIS tool by a health worker was influencing patients, for example, filling out of home‐based records (HBR) (e.g. Essen 1994; Tarwa 2007) or follow up with patients for treatment adherence (Da Costa 2009; Leon 2015). In two studies, district managers were the participating subjects. In 12 other studies, participants referred to a combination of those mentioned above.

Sixteen of the 50 included studies had mother and child health care as the main health care area. The setting was general practice without a specific focus in 10 studies. Three studies focussed on infectious diseases in general; two studies on cancer care, two on family health, two on hypertension and two on diabetes. Health financing, HIV/Aids, Malaria, tuberculosis and reproductive health were the main topics in one study each. A combination of different health areas other than maternal and child health was found in two studies. In three studies, the health subject was not clearly identifiable. The HIS was exclusively addressed in another three studies.

Thirty‐six studies focused on an information tool; seven studies tackled data quality issues; and another seven focussed on a variety of health information issues.

We report the quality of studies by criteria (Table 3) and also the distribution of overall quality scores (Table 4). The larger group of studies had minor to moderate quality concerns, for example, not reporting how findings were integrated into the existing body of evidence or confounders not taken into account (17 studies). Seven studies had moderate to serious quality issues. The main reasons were lack of adequate description of the sampling or recruitment strategy (7), a lack of clearly identifiable outcomes (6), not taking confounders into account (7), a weak description of the analysis performed (5) and no integration of findings into the context of other research (7). The full results of the quality assessment for each of the included studies can be found in Supplementary file 5.

**TABLE 3:** Quality assessment of included studies

Quality criteria	N	%
Aim described	47	94
Context described	47	94
Method described and adequate	43	86
Sampling described	21	42
Outcome described	36	72
Confounders described	11	22
Analysis described	24	48
Findings described	43	86
Integration of findings	30	60
Ethical considerations described	26	52
No other concerns	21	42

**TABLE 4:** Frequency distribution of quality assessment scores

Level	Score	Number of studies
No concerns	11	0
Minor concerns	9, 10	14
Moderate‐minor	7, 8	17
Moderate concerns	5, 6	12
Moderate‐serious	2–4	7
Serious concerns	1	0
**Mean score**	**7**	**50**

### 3.2 Mapping decision‐making components in the literature

Twenty‐nine studies focussed on decision‐making at the PHC facilities; two examined decision‐making at the district level of the PHC system. Seven studies focussed at several health care levels. In 12 studies, the setting was not clearly identifiable.

The 35 studies addressing decision‐making at the PHC facilities examined the use of electronic health record systems (7), decision algorithms for disease management (6), HBR (5), factors related to the use of electronic health technology in general (4), the partograph (3), the use of paper‐based medical records (5), SMS reminders for patient follow‐up (2), mobile cameras for screening and treatment (2) and the use of a communication guide for nurses (1). The two studies conducted at district level focussed on decision‐making in the frame of an assessment of WHO district HIS (DHIS) and in an evaluation of the district health management information system.

Combinations of different decision levels were found in 13 studies. In one study, the use of a primary care information system for planning and evaluation of actions at health facility and district level was examined. Eight studies compared the registry entries from the health facilities with the DHIS in the frame of data quality assessments. Single studies focussed on the following issues: the applicability of a basic information system for the planning of work of nurses in PHC facilities and for planning of family health activities at the higher levels; influencing factors on the intention of PHC physicians to collect data exhaustively in medical registries; challenges on health facility and district levels of implementing an Integrated Disease Surveillance and Response strategy using the current HMIS; reasons for under‐reporting of notifiable communicable diseases; and factors related to the PHC facilities, higher levels as well as policy and planning.

Different types of decision‐making were identified. Clinical decisions were most often addressed (19 studies). They were related to diagnosis and treatment (4), treatment only (4) and management of deliveries (5). In three studies it was not clear what type of clinical decisions were considered. Two studies indirectly addressed clinical decision‐making in the area of growth and development monitoring of children. One study was about diagnosis only.

Four studies focussed on managerial decisions exclusively: two addressed evaluation and planning of family health activities; one considered the implementation of HIS policy and objectives, technical feasibility, financial and political viability, administrative operability, internal quality of information, external resources, managerial support and systems impact; and one mentioned managerial decisions without any clear specification.

One study reported on public health decisions only, defined as the identification of strategies and interventions in the context of integrated disease surveillance.

In 13 studies, a combination of different types of decisions was mentioned and in another 13 studies, the decision type was not clearly identifiable. By focussing on the content of the different types of decisions, we found that more tasks were related to managerial decisions than clinical and public health decisions.

Regarding the tools supporting decision‐making, 21 studies examined paper‐based decision support tools. Six studies examined digital and five studies explicitly named computers as decision supporting tools. A combination of tools was found in 13 studies. In five studies, the support tool was not clearly identifiable.

When mapping the decision‐making components in the literature, we found that the single component most frequently found was ‘considering the outcomes’, which refers to the recording practices documenting the results of clinical or public health events. Transforming information and reporting were found on 21 and 20 occasions, respectively and are activities that tend to go together (i.e. the fact of reporting often entails a previous data transformation process). Twenty studies considered information to assess a situation or problem. The component ’best course of action’ was addressed in 16 studies and ‘knowledge’ in 14. Information used for follow‐ups was retrieved in 11 studies. Much less frequently found components included those that may entail mechanisms to improve the system (e.g. auditing technical guidance) and communication. The least frequently found related to the aids to health workers or decision‐makers to better understand and cope with the HIS itself (Table 5).

**TABLE 5:** Decision‐making components mapped in the research literature

Component	Number of studies
A.3 considering the outcomes of the decisions	24
B.5 transforming information	21
B.6 Reporting information	20
A.1 Assessing a situation	20
A.2 Choosing the best course of action	16
C.8 Acquiring and using knowledge	14
A.4 Following up the cases or situations	11
D.11 Auditing data quality	5
C.7 Providing technical guidance	5
C.9 Auditing health care	4
C.10 Communicating between health care actors	4
D.12 Helping to cope with the HIS	2

Looking at the overall categories, (A) information for immediate decision‐making components were found 71 times, (B) information to document health care events 41, (C) to maintain quality of care 27, and (D) related to use of the information system 7 times.

### 3.3 Information use for decision‐making

The 14 studies with minor quality concerns were further examined to understand how information was used in decision‐making, as well as barriers and opportunities for the use of information. We extracted in total 429 statements.

Eight studies examined paper‐based information tools (Abud and Gaviá 2015; Alberti 2006; Amoakoh‐Coleman 2015; Ly 2015; Mahmood and Ayub 2010; Palombo 2014; dos Santos Ribeiro Silva 2013; Wakgari 2015). Three studies were conducted in Brazil (Palombo 2014; Abud and Gaviá 2015; dos Santos Ribeiro Silva 2013). The other studies were conducted in Tunisia (Alberti 2006), Pakistan (Mahmood and Ayub 2010), Ghana (Amoakoh‐Coleman 2015), Ethiopia (Wakgari 2015) and Mali (Ly 2015). Health care areas included child and maternal health (Palombo 2014; Abud and Gaviá 2015; Wakgari 2015; Amoakoh‐Coleman 2015), namely HBR (Palombo 2014; Abud and Gaviá 2015; dos Santos Ribeiro Silva 2013) and partograph (Wakgari 2015); general practice (Ly 2015; Mahmood and Ayub 2010), family health (dos Santos Ribeiro Silva 2013) and diabetes management (Alberti 2006).

Three studies focussed on electronic information tools; two of these in Israel (Margalit 2006; Shachak 2009) and the other one was done in Mexico (Doubova 2013). Studies reported on electronic medical records in different health care areas, in general PHC practice, in diabetes care and management.

Three studies which reported on a combination of tools were carried out in the state of Hawaii of the United States (Tseng 2010), in Bahrain (Al‐Hashimi 2014) and in South Africa (Leon 2015). These studies examined the use of different health technologies, such as the Internet, electronic medical records, e‐prescribing and Personal Digital Assistants in the area of health financing (Tseng 2010), the use of a computer‐based SMS treatment adherence tool for the management of hypertension (Leon 2015), as well as the use of paper and electronic disease notification tools in the area of infectious diseases (Al‐Hashimi 2014).

The majority of the studies included in the in‐depth analysis were conducted in PHC settings. Two other studies took place in mixed health care settings (Wakgari 2015; Amoakoh‐Coleman 2015) and in three studies the setting could not be clearly identified (Tseng 2010; Doubova 2013; Ly 2015).

The main thematic areas reported by studies were:

(A) Immediate decision‐making
  1. Use for immediate decision making (e.g. recording)
    - Associated factors with recording (Palombo 2014; Abud and Gaviá 2015; Ly 2015; Alberti 2006).
  2. Documenting health care events
    ‐ Associated factors with reporting of information for notifiable, communicable diseases (Al‐Hashimi 2014);
    ‐ Association between the use of electronic information tools and knowledge regarding drug costs (Tseng 2010).
(B) Documenting health care events
  - Data transfer from lower to higher reporting levels (Amoakoh‐Coleman 2015).
(C) Maintaining quality of care
  - Sources of knowledge used for decision‐making (Tseng 2010);
(D) Use of the information system
  ‐ Experience of health workers with an electronic medical record system (Shachak 2009).
  ‐ Time spent with information tool per patient visit and correlated factors (Margalit 2006);
  ‐ Factors associated with the use of information tools like the partograph: health facility characteristics, profession, qualification, on‐the‐job training, knowledge and attitudes of health workers (Wakgari 2015);
  ‐ Experience of health workers with a paper‐based communication tool (dos Santos Ribeiro Silva 2013);
  ‐ Effects on the patient health worker interactions while using a follow‐up tool (Leon 2015);
  ‐ Knowledge of health workers regarding reporting (Mahmood and Ayub 2010);
  ‐ Type of information tool used in clinical care (Tseng 2010; Wakgari 2015);
  ‐ Level of utilisation of information tool (Tseng 2010; Margalit 2006);
  ‐ Knowledge of health workers regarding recording of information (Mahmood and Ayub 2010); knowledge of patients/caretakers (Palombo 2014).
  ‐ Tasks for which the information tool was used (Margalit 2006; Shachak 2009; Doubova 2013).

### 3.4 Use of information: Barriers and opportunities

Barriers and challenges for the use of information were linked to the health system, the information tools used, to health managers and to health workers. Box 1 summarises the key messages.

**Box 1: Barriers in the use of information**

**Table:** 

Health system	Weak policy and programme implementation
	Unavailability of information tools
	Political, technical and infrastructural restrictions for accessing information
	Resource constraints
Information tools	Impracticability of paper‐based information tools
	Negative impact of electronic information tools on communication and patient safety
	Influence of factors outside the reach of information tools
Health managers	Lack of feedback to and supervision of health workers
Health workers	Fears of health workers (regarding new tools)
	Disparities in health workers' knowledge about procedures and responsibilities, and differences in health workers' attitudes
	Difficulties in building trust‐ and respectful relations with patients

Major barriers from the health system for the use of information in PHC were identified in terms of weak political strategies and health programmes that fail to have an impact at the frontline (Palombo 2014; Abud and Gaviá 2015; Mahmood and Ayub 2010) or that do not take into account the prevalence of diseases when planning health care activities, as suggested in the case of hypertension management (Doubova 2013); or when the public health services are dysfunctional and non‐responsive (Leon 2015). The failure of the health systems to guarantee the provision and availability of information tools to health workers was found twice (Abud and Gaviá 2015; Al‐Hashimi 2014).

For example, in terms of political, infrastructural as well as technical restrictions regarding the availability and usability of information, a study from Hawaii describes the following:

*“At the time of our study, an informal review indicated that several health plans in Hawaii made co‐payment and formulary information available*via *the Internet, but not necessarily* via *EHR [Electronic Health Record], e‐prescribing, or PDA [Personal Digital Assistant]. […].A highly promoted type of e‐prescribing software in Hawaii contained formulary information only for the single health plan that sponsored its adoption. […] This study indicates that improving physicians' knowledge of drug costs will require more than simply increasing physicians' use of health IT”* (Tseng 2010).

A study in Ethiopia (Wakgari 2015) identified a lack of human resources as a major barrier for HIS. And a study in Tunisia highlighted resource constraints:

*“[…] structured care with computerised recall improves the process of care but many countries do not have the luxury of computers in primary care”*(Alberti 2006).

For paper‐based information systems, the existence of numerous registers and entry forms, the manual data collection (i.e. handwriting of forms), the volume of data items recorded as well as a lack of internal quality control mechanisms together with a generally high patient load in PHC facilities were identified as major obstacles (Mahmood and Ayub 2010; Amoakoh‐Coleman 2015); the study in Pakistan found:

*“[…] pressure of service provision and at the same time recording a single event in at least two to three different places might result in illegible, inaccurate records and missing entries”*(Mahmood and Ayub 2010).

Additionally, the use of general medical record registers not linked to individual patients was considered as a problem for following up the history and treatment progress (Amoakoh‐Coleman 2015). In the case of HBR, difficulties with the technical contents of the tool (e.g. reference curves in z‐scores and body mass index chart by age) were considered as built‐in barriers for its use (Palombo 2014; Abud and Gaviá 2015). In a study in Ethiopia examining the reasons for not using the partograph, it was not clear whether other tools to monitor labour, such as clinical records, monitoring charts or simple pieces of paper were used because of the difficulties of the content of the partograph or because of its unavailability (Wakgari 2015).

Studies looking at electronic information systems mentioned as barriers for their use the occurrence of missing data and non‐plausible values as well as a lack of quality of health care indicators incorporated in the tools:

*”Also, the information in the EHR [Electronic Health Record] did not allow learning about some estimators of quality of care related to health outcomes, such as the level of adherence to treatment and about the quality of life. This information is not registered routinely in the EHR" (Mexico)*(Doubova 2013).

A study in Israel mentioned as a barrier of electronic information systems their strong focus on technical functionality and interoperability at the expense of their usability. The study reported on the occurrence of “new types of medical errors”, such as:

*“adding information to the wrong patient's chart, and unintentionally selecting an erroneous item (diagnosis or medication) from a scroll‐down list located above or below the desired item”*(Shachak 2009)

with potential effects on patients' safety. Another challenge related to electronic information systems was reported as “cognitive overload” (Margalit 2006) of health care providers, occurring when data had to be entered into the electronic system at the same time a patient was present and had to be cared for. Electronic information systems were also described as negatively affecting patient‐centeredness:

*“This study suggests that the way in which physicians use computers in the examination room can negatively affect patient‐centred practice by diminishing dialogue, particularly in the psychosocial and emotional realm […] Screen gaze appears particularly disruptive to psychosocial inquiry and emotional responsiveness, suggesting that visual attentiveness to the monitor rather than eye contact with the patient may inhibit sensitive or full patient disclosure” (Israel)*(Margalit 2006).

Issues related to health managers were also categorised as barriers towards the use of information in PHC (Mahmood and Ayub 2010; Ly 2015; Al‐Hashimi 2014; Tseng 2010; Wakgari 2015; dos Santos Ribeiro Silva 2013; Amoakoh‐Coleman 2015). Lack of feedback from higher health system authorities to health facility workers was several times described as a demotivating factor for health workers (Mahmood and Ayub 2010; Ly 2015; Al‐Hashimi 2014). Also the large amount of administrative and managerial responsibilities, the participation of health workers in many events other than clinical care and the low income and poor working conditions were mentioned as barriers attributable to management (Ly 2015). Two studies mentioned weak supervisory mechanisms (Mahmood and Ayub 2010; Ly 2015). Other critical points were the existence of impractical reporting and notification procedures (Al‐Hashimi 2014; Doubova 2013; Amoakoh‐Coleman 2015) and a lack of standardised procedures to evaluate information systems:

*“At the time of conducting this study, the […] Centre for Health Information Systems […] had introduced data quality audits in 2011 but had not yet developed Standard Operating Procedures (SOPs) for data management, nor defined the level of accuracy and completeness that should be achieved. It was therefore unclear as to what acceptable standards of data quality are […]” (Ghana)*(Amoakoh‐Coleman 2015).

Several studies described challenges related to health workers (dos Santos Ribeiro Silva 2013; Mahmood and Ayub 2010; Shachak 2009; Ly 2015; Doubova 2013; Palombo 2014; Abud and Gaviá 2015; Al‐Hashimi 2014; Tseng 2010; Wakgari 2015). Two studies reported fears of health workers regarding the use of information tools (dos Santos Ribeiro Silva 2013; Mahmood and Ayub 2010), but for different reasons. The implementation of a new tool was considered as a new practice interfering with the daily routine of health workers, causing "anxiety and uneasiness" (dos Santos Ribeiro Silva 2013). Difficulties in the use of key components and differences in the level of experiences of health workers with the new tools made them "feeling insecure about the challenge of introducing an innovative procedure into their established routines". Concerns about "the time spent for exercising this new practice" and the feeling of work overload were also found (dos Santos Ribeiro Silva 2013). Health workers' fears related to the use of information tools were also rooted in the perception of these tools as employee controlling systems:

*“Another factor which creates fears among health workers in compiling and reporting data is that information systems could monitor employee's work and that repercussion will occur if the employees will not attain a particular level on a performance indicator” (Pakistan)*(Mahmood and Ayub 2010).

It has to be noted that both studies addressing fears of health workers were related to paper‐based information tools.

Another health worker‐related challenge mentioned was the individual variation in the ease of use of information tools (Shachak 2009) and the existing differences in health workers' knowledge and attitudes (Al‐Hashimi 2014; Palombo 2014; Abud and Gaviá 2015; Wakgari 2015; Ly 2015; Doubova 2013; Mahmood and Ayub 2010; Amoakoh‐Coleman 2015). On the one hand, the level of ease in the use of information tools was highly dependent on the individual health worker's style, experience and communication skills, and not so much on formal accreditation criteria (Shachak 2009). On the other hand, other studies pointed at the importance of more formal training, suggesting that the lack of continued medical education to change physicians' practice of recording, prescribing, screening, counselling and guideline adherence was an obstacle for an improved use of information (Ly 2015; Doubova 2013). Differences in health workers' knowledge regarding the recording and reporting practice (Mahmood and Ayub 2010), regarding procedures, communication channels and responsibilities for the notification of infectious diseases (Al‐Hashimi 2014) and regarding the utilization or non‐utilization of a standardized labour monitoring tool (Wakgari 2015) have also been reported. Some attributes and attitudes of health workers were also considered problematic (Amoakoh‐Coleman 2015; Ly 2015; Al‐Hashimi 2014; Palombo 2014; Abud and Gaviá 2015). Attributes of the staff entering data into electronic information systems, such as the number of years a health worker is in practice (Ly 2015) or the forgetfulness of the health worker (Amoakoh‐Coleman 2015; Al‐Hahimi‐2014) were reported. Additionally, a lack of conscientiousness, appreciation and commitment of the health workers towards the practice of recording of information (Palombo 2014; Abud and Gaviá 2015) was of concern.

Box 2 lists key opportunities for the use of information for decision‐making as found in the included studies.

**Box 2: “Opportunities for the use of information for decision‐making”**

**Table:** 

Health system	Well‐implemented health programmes (e.g. vaccination) create positive environment for the use of information
Benefits of electronic information systems	Internal quality
	Efficiency
	Inclusion of knowledge and action items
Benefits of paper‐based information systems	Use in resource constraint settings
	Alternative forms to improve usability
Communication	Time for patients
	Increased knowledge of health workers and patients
	Satisfaction of health workers
Health workers	General openness of health workers to test and use information tools in practice.

From the health systems perspective, opportunities for the use of information in PHC were mainly seen in strong national programmes (Alberti 2006; Palombo 2014; Abud and Gaviá 2015), particularly in vaccination programmes:

*“Moreover, the National Programme of Immunisation has a continued development of structures for human resources, surveillance and supervision of activities in vaccination rooms that standardise the procedures and bring stability to the actions directed at vaccination” (Brazil)*(Palombo 2014).

Within such a stable programmatic environment, improvements in the use of information could be achieved, as it was mentioned in the case of HBR being used by health workers (Palombo 2014; Abud and Gaviá 2015). Opportunities within the HIS were identified for electronic (Tseng 2010; Margalit 2006; Shachak‐2009; Doubova 2013; Amoakoh‐Coleman 2015) as well as for paper‐based information systems (Amoakoh‐Coleman 2015; Alberti 2006; dos Santos Ribeiro Silva 2013).

One opportunity related to electronic information systems was their potential to share information over different channels and media (Tseng 2010). The efficiency of electronic systems was mentioned in several studies (Tseng 2010; Amoakoh‐Coleman 2015; Margalit 2006; Shachak 2009). The possibility to integrate internal checks to assure data quality (Amoakoh‐Coleman 2015; Doubova 2013) and the option of integrating action and knowledge items to support clinical decision‐making (Tseng 2010) were highlighted in those studies. Minimising recall errors of health care providers through the ease of reviewing patients' histories and test results, the opportunity to include and educate patients with the help of a computer and finally anticipated improvements in the quality of patient care were advantages of electronic systems (Margalit 2006; Shachak 2009; Doubova 2013).

The patient and quality of care monitoring components of electronic information systems were mentioned in Doubova 2013 and Amoakoh‐Coleman 2015. Electronic systems were also seen as an opportunity to increase the available time for patient‐health worker communication, because less time is spent during consultations for manual tasks (Shachak 2009).

For paper‐based information systems, the implementation of disease‐specific records was seen as an opportunity, especially in resource‐limited settings (Alberti 2006); also the creation of alternative paper‐based forms to ease the recording and counting of information for reporting (Amoakoh‐Coleman 2015). The possibility of integrating ‘assessment’ and ‘communication’ components into paper‐based tools was mentioned as an opportunity to increase the range of available information for health workers with a potential positive impact on the health worker as well as patient knowledge (dos Santos Ribeiro Silva 2013).

A general openness of health workers to test new information tools (dos Santos Ribeiro Silva 2013) as well as a good knowledge base and positive attitudes of care providers towards information tools were described as opportunities (Tseng 2010; Mahmood and Ayub 2010; 1223‐Wakgari 2015).

In the context of child and maternal health care, it was suggested that information obtained in consultations should not only improve the general quality of care but even function as enabler of equity in health care, because equally recorded information can improve patient outcomes (Abud and Gaviá 2015). Other components of information systems should be used to inform health care providers, to obtain epidemiological information, to measure performance of health care teams and to conduct further research (Doubova 2013; Palombo 2014). Health care providers should not blindly record information, but ideally use this information for communication (dos Santos Ribeiro Silva 2013; Abud and Gaviá 2015), patient education (dos Santos Ribeiro Silva 2013; Margalit 2006; Shachak 2009) and further actions (Abud and Gaviá 2015; Amoakoh‐Coleman 2015). The use of information should not only strengthen health care workers but also patients and caregivers in their decision‐making roles: through information, patients ideally participate in their own care (Margalit 2006). Closely linked with the description of the ideal use of information was the extension of responsibilities of health workers, health managers and patients and caregivers. Active participation in, commitment to and appreciation of the practice of recording of information was required from health workers, health managers but also from patients and caregivers (Palombo 2014; Abud and Gaviá 2015). Health care workers should perceive their role as guides, controllers and instructors for patients, to enable further engagement of patients into their own care (Palombo 2014; Abud and Gaviá 2015).

## 4 DISCUSSION

We conducted a framework synthesis of the research literature in order to inform the development of an innovative paper‐based HIS (PHISICC), having as a reference a list of twelve decision‐making components resulting from the characterisation of HIS in three African countries. Across all included studies, we could not retrieve any additional decision‐making component beyond those that we had already identified (Table 1), which suggests that our framework fits the current research literature, in terms of HIS use for frontline health workers' decision‐making.

Indeed, the bulk of included studies addresses or mentions in one way or another most of the decision‐making components and most of them are limited to specific health care areas (as opposed to the whole system). However, when we looked in‐depth in a selection of studies, these tended to focus on the technicalities of information systems (e.g. how digital systems run) rather than on their use for decision making (e.g. how digital systems might lead to better or more timely diagnoses).

The technical focus of most findings is consistent with requests to health workers to periodically synthesise and ‘report’ their activities and resource consumption to higher levels of the health system, where information is aggregated and forwarded to national^13^, ^14^ and international levels.^15^

Data quality is a recurrent issue in international health and development: twenty years ago it was already defined in terms of relevance, completeness, timeliness and accuracy^16^; 10 years later, the criteria were similar^2^ including, for example, consistency and validity^17^; even recent evidence suggests that the focus on data quality has hardly changed.^18^ It is striking that the actual use of data and how data and information relates to decision‐making is much less studied. These seems to be consistent with other research evidence. For example, a recent scoping review^19^ focussed on intervention studies that aimed to improve data quality and use within RHIS in low‐ and middle‐income countries. The review found that a combination of interventions, addressing both behavioural and technical factors, improved data quality and use. Another systematic review^20^ looked at challenges associated with the use of data from RHIS in low‐ and middle‐income countries. The review found that the challenges most frequently addressed (or reported) were of technical nature.

In times when recommendations on digital health are increasingly widespread,^21^ donors, partners and countries should take note of the numerous design and implementation challenges that digitalisation may bring, as illustrated in some of our examples in this review and elsewhere.^22^ Paper‐based systems, on the other hand, also have caveats, such as the error‐proneness of writing and transcribing information. Some challenges are strikingly basic (e.g. the need for planning or training), while others reach beyond the domain of health (e.g. equipment and infrastructures).

Decision‐making is indeed a complex process, where data and evidence is just one of the components. Barriers and facilitators to the use of evidence have been described in the literature for years across several settings.^23^, ^24^ Most studies in this review point at technical or managerial challenges but some studies also reported on the need to address “human factors” in the interaction with HIS; for example, building trustful and respectful relations with patients, taking into account psychosocial factors or the needs of health care provision. These ‘human factors’ reach further and are consistent with findings in other related reviews.^25^

Even though data use for decision‐making is widely mentioned across the literature, the in‐depth analysis could not reveal any substantial insight or example on how HIS should be tailored to improve decision‐making, either in clinical care (e.g. quality of care), public health (e.g. coverage of preventive measures) or health care management (e.g. resource forecasts). The interplay between data and decision‐making is hardly studied; and where it is, it offers disappointing findings.^26^

### 4.1 Limitations of this framework synthesis

Despite having followed standard systematic review methods, our framework synthesis has several limitations, some of them deriving from the diverse level of detail in the included studies. We may have left out some relevant studies, although we have experienced a certain degree of saturation and repetition in the terms and concepts reported and completeness in relation to the components that emerged from the preceding field work. Furthermore, included studies are not necessarily rooted into a particular HIS framework which may lead to inconsistencies in the terminology, definitions or in the understanding of HIS in different settings. We acknowledge that our search strategy may become out of date; however, this framework synthesis was done in order to inform the ideation of a paper‐based HIS intervention (PHISICC), which it did; and on the other hand, we believe that the studies published later on would hardly change our findings as suggested by the evidence found in more recent reviews.^19^, ^20^ Lastly, we did not register the protocol of this review. Open access funding provided by Universitat Basel.

## 5 CONCLUSIONS

Our framework synthesis of available research revealed a strong focus on the technicalities of recording and reporting of information to higher levels of the health system, rather than on decision‐making.

The twelve decision‐making components developed during field work in Côte d'Ivoire, Mozambique and Nigeria proved to be comprehensive and serve as a guide to design innovative paper‐based HIS (PHISICC), which are more user‐friendly, facilitating decision‐making.

## CONFLICT OF INTEREST

The authors declare that they have no conflict of interest.

## AUTHOR CONTRIBUTIONS

All authors substantially contributed to conception and/or development of the study and the manuscript. All authors read and approved the final manuscript.

## ETHICS STATEMENT

No ethical review was required for this work (only published papers were used).

## Supporting information

Supplementary Material

Supplementary Material

Supplementary Material

Supplementary Material

Supplementary Material

## Data Availability

All relevant data are within the manuscript and its Supporting information files, including the data extraction and synthesis workbook. The protocol can be accessed through the corresponding author.
