# Supplementary material for: Framework synthesis to inform the ideation and design of a paper‐based health information system (PHISICC)
Source: Int J Health Plann Manage. 2022 Apr 23;37(4):1953–72. doi: 10.1002/hpm.3487 (PMC9544999; doi:10.1002/hpm.3487)
Supplement: Supplementary file 2 — Supplementary Material [file HPM-37-1953-s004.docx]

**Supplementary file 2: Search Strategies**

*Article title*: Research on Health Information Systems Focus on Technical Aspects Rather than on Decision-Making. A Framework Synthesis to Inform the Ideation of Paper-based Health Information Systems (PHISICC).

*Journal name*: IJHPM

*Authors information including author names, affiliation, and email address of the corresponding author*:

Meike-Kathrin Zuske^1, 2^, Christian Auer^1, 2^, Sandy Oliver^3,4^, John Eyers^5^, Xavier Bosch-Capblanch^1, 2^ ^*^

^1^ Swiss Tropical and Public Health Institute, Basel, Switzerland;

^2^ University of Basel, Basel, Switzerland

^3^ University College London, EPPI-Centre, Social Research Institute, London, United Kingdom

^4^ University of Johannesburg, Africa Centre for Evidence, Faculty of Humanities, Johannesburg, South Africa

^5^ Independent Consultant & Senior Research Fellow, 3ie, c/o LIDC, 20 Bloomsbury Square, London WC1A 2NS, United Kingdom

^*^ Correspondence: [x.bosch@unibas.ch](mailto:x.bosch@unibas.ch)

**Search Strategies – Health Information Systems in LMICs**

1. **Ovid MEDLINE(R) In-Process & Other Non-Indexed Citations, Ovid MEDLINE(R) Daily and Ovid MEDLINE(R) <1946 to Present>**

1 exp Community Health Services/ (259495)

2 Primary Health Care/ (59410)

3 General practice/ or Community mental health services/ or Family practice/ or Home care services/ or Family physicians/ or Community pharmacy services/ or Preventive health services/ (139858)

4 Community Health Aides/ (3682)

5 Allied Health Personnel/ (10479)

6 Midwifery/ (16256)

7 Voluntary Workers/ (8201)

8 Community Health Nursing/ (18806)

9 (primary care or primary health care or primary healthcare or primary medical care or community health or rural health or district health).tw. (116009)

10 ((community or family or general) adj2 (practice or practitioner* or physician* or doctor*)).tw. (99005)

11 (community adj2 (mental health or psychiatr* or care or pharmac*)).tw. (20388)

12 preventive health service*.tw. (543)

13 (((community health or village health) adj3 (aide? or worker$)) or barefoot doctor?).tw. (2815)

14 (allied health personnel or allied health worker? or paramedical personnel or paramedics).tw. (3728)

15 (birth attendant$ or midwif$ or mid-wif$ or midwiv$ or mid-wiv$ or traditional birth or doula$).tw. (19793)

16 (voluntary worker? or volunteer worker?).tw. (155)

17 (lay adj (health work$ or health personnel)).tw. (182)

18 or/1-17 (533180)

19 Developing Countries/ (65459)

20 (less-developed countr$ or third world countr$ or under developed countr$ or underdeveloped countr$ or developing nation? or less developed nation? or third world nation? or under developed nation? or underdeveloped nation? or developing countr$).ti,ab. (48869)

21 (low income count$ or low income nation? or middle income count$ or middle income nation? or "low and middle income" or lmic or lmics).ti,ab. (10013)

22 exp Africa/ (207107)

23 exp Asia, Western/ (209136)

24 exp Asia, Southeastern/ (73539)

25 exp Asia, Central/ (6383)

26 exp caribbean region/ or exp central america/ or latin america/ or mexico/ or exp south america/ or borneo/ or exp indian ocean islands/ or indonesia/ or pacific islands/ or exp melanesia/ or exp micronesia/ or exp polynesia/ or philippines/ or prince edward island/ or exp west indies/ or oceania/ (224728)

27 or/19-26 (736419)

28 Epidemiologic studies/ (7031)

29 exp case control studies/ (765481)

30 exp cohort studies/ (1511505)

31 (cohort adj (study or studies)).tw. (112086)

32 Cohort analy$.tw. (4640)

33 (Follow up adj (study or studies)).tw. (40616)

34 observational study/ (19528)

35 (observational adj (study or studies)).tw. (58786)

36 Longitudinal.tw. (164655)

37 Retrospective.tw. (334066)

38 Cross sectional.tw. (212610)

39 Cross-sectional studies/ (210207)

40 ((Ecologic* or Panel) adj (study or studies)).tw. (5148)

41 or/28-40 (2152049)

42 randomized controlled trial.pt. (411144)

43 controlled clinical trial.pt. (90371)

44 randomi?ed.ab. (407543)

45 placebo.ab. (168016)

46 drug therapy.fs. (1835212)

47 randomly.ab. (245501)

48 trial.ab. (352518)

49 groups.ab. (1533468)

50 ((before adj5 after) or (pre adj5 post) or pretest or pre test or posttest or post test or quasiexperiment* or quasi experiment* or time series or time point?).ti,ab. (447346)

51 or/42-50 (3987026)

52 data collection/ or data accuracy/ or datasets as topic/ or focus groups/ or health impact assessment/ or interviews as topic/ or lot quality assurance sampling/ or narration/ or records as topic/ or registries/ or "surveys and questionnaires"/ or vital statistics/ (535442)

53 medical informatics/ (9114)

54 health information exchange/ (240)

55 nursing informatics/ (1090)

56 public health informatics/ (1112)

57 dental informatics/ (156)

58 medical informatics applications/ (2162)

59 medical records/ (63042)

60 medical record linkage/ (3986)

61 medical record systems, computerized/ (18504)

62 electronic health records/ (9578)

63 nursing records/ (6355)

64 ambulatory care information systems/ (1162)

65 medical order entry system/ (1714)

66 decision support systems, management/ (927)

67 registries/ (61585)

68 information management/ (3287)

69 computer communication networks/ (12933)

70 ((medical or clinical or nursing or dental or public health or community health or rural health or electronic or computer*) adj3 (data or informati* or record or records or network* or registr*)).ti,ab. (275100)

71 exp health information management/ (638)

72 systems analysis/ (4739)

73 systems integration/ (8693)

74 health information systems/ (539)

75 management information systems/ (3615)

76 "personnel staffing and scheduling information systems"/ (421)

77 ((health or management or medical or clinical) adj3 (system or systems or information)).ti,ab. (168169)

78 or/52-77 (989398)

79 exp Hospitals/ (232173)

80 exp Tertiary Care Centers/ or exp Tertiary Healthcare/ (4643)

81 (hospital* or tertiary).ti,ab. (998843)

82 review.pt. (2086053)

83 meta analysis.pt. (63326)

84 (systematic review or literature review).ti. (71678)

85 news.pt. (175475)

86 comment.pt. (656885)

87 editorial.pt. (397836)

88 cochrane database of systematic reviews.jn. (12142)

89 comment on.cm. (656885)

90 or/79-89 (4206386)

91 exp animals/ (19986071)

92 humans/ (15775585)

93 91 not (91 and 92) (4210486)

94 18 and 27 and 51 and 78 (3815)

95 94 not (90 or 93) (2959)

96 18 and 27 and 41 and 78 (3699)

97 96 not (90 or 93) (2647)

1. **Embase**

1 exp community care/ (105065)

2 primary health care/ or primary medical care/ (123197)

3 general practice/ (72264)

4 community mental health/ (2692)

5 home care/ (50951)

6 general practitioner/ (69788)

7 pharmacy/ (61256)

8 preventive health service/ (23458)

9 health auxiliary/ (3909)

10 paramedical personnel/ or health visitor/ or exp midwife/ or exp nurse/ or nursing assistant/ or paramedical profession/ or pharmacist/ (214971)

11 midwife/ or nurse midwife/ (25757)

12 voluntary worker/ (5492)

13 community health nursing/ or community psychiatric nursing/ (25938)

14 (primary care or primary health care or primary healthcare or primary medical care or community health or rural health or district health).tw. (143446)

15 ((community or family or general) adj2 (practice or practitioner* or physician* or doctor*)).tw. (121015)

16 (community adj2 (mental health or psychiatr* or care or pharmac*)).tw. (27590)

17 preventive health service*.tw. (589)

18 (((community health or village health) adj3 (aide? or worker$)) or barefoot doctor?).tw. (3015)

19 (allied health personnel or allied health worker? or paramedical personnel or paramedics).tw. (4809)

20 (birth attendant$ or midwif$ or mid-wif$ or midwiv$ or mid-wiv$ or traditional birth or doula$).tw. (21414)

21 (voluntary worker? or volunteer worker?).tw. (143)

22 (lay adj (health work$ or health personnel)).tw. (200)

23 or/1-22 (708118)

24 developing country/ (81812)

25 (less-developed countr$ or third world countr$ or under developed countr$ or underdeveloped countr$ or developing nation? or less developed nation? or third world nation? or under developed nation? or underdeveloped nation? or developing countr$).ti,ab. (59023)

26 (low income count$ or low income nation? or middle income count$ or middle income nation? or "low and middle income" or lmic or lmics).ti,ab. (11917)

27 exp Africa/ (245485)

28 exp Asia/ not Japan/ (610051)

29 exp caribbean/ or exp caribbean islands/ (25427)

30 "south and central america"/ or exp central america/ or exp south america/ or mexico/ (196128)

31 exp Indian Ocean/ (3502)

32 exp Pacific islands/ (33933)

33 or/24-32 (1107664)

34 Clinical study/ (75398)

35 Case control study/ (102975)

36 Family study/ (11244)

37 Longitudinal study/ (85925)

38 Retrospective study/ (454442)

39 Prospective study/ (326919)

40 Randomized controlled trials/ (94000)

41 39 not 40 (324252)

42 Cohort analysis/ (235935)

43 (Cohort adj (study or studies)).mp. (160829)

44 (Case control adj (study or studies)).tw. (92148)

45 (follow up adj (study or studies)).tw. (49199)

46 (observational adj (study or studies)).tw. (88401)

47 (epidemiologic$ adj (study or studies)).tw. (82773)

48 (cross sectional adj (study or studies)).tw. (115757)

49 ((Ecologic* or Panel) adj (study or studies)).tw. (5487)

50 observational study/ (87166)

51 or/34-38,41-50 (1506256)

52 Clinical trial/ (854977)

53 Randomized controlled trial/ (396525)

54 Randomization/ (69733)

55 Single blind procedure/ (21768)

56 Double blind procedure/ (127154)

57 Crossover procedure/ (46530)

58 Placebo/ (272440)

59 Randomi?ed controlled trial$.tw. (132157)

60 Rct.tw. (19726)

61 Random allocation.tw. (1499)

62 Randomly allocated.tw. (24226)

63 Allocated randomly.tw. (2095)

64 (allocated adj2 random).tw. (750)

65 Single blind$.tw. (17087)

66 Double blind$.tw. (160050)

67 ((treble or triple) adj blind$).tw. (532)

68 Placebo$.tw. (229534)

69 Prospective study/ (326919)

70 ((before adj5 after) or (pre adj5 post) or pretest or pre test or posttest or post test or quasiexperiment* or quasi experiment* or time series or time point?).ti,ab. (604588)

71 or/52-70 (2056957)

72 Case study/ (37052)

73 Case report.tw. (302295)

74 Abstract report/ or letter/ (954301)

75 or/72-74 (1286764)

76 71 not 75 (2011657)

77 medical information system/ (16136)

78 information system/ or clinical data repository/ or exp computerized provider order entry/ or decision support system/ or expert system/ or financial information system/ or geographic information system/ or medical information system/ or nursing information system/ or online system/ or exp performance measurement system/ or reminder system/ (103168)

79 bioinformatics/ or dental informatics/ or medical informatics/ or nursing informatics/ (48284)

80 data processing/ or clinical data repository/ or data synthesis/ or data analysis/ or electronic data interchange/ or data collection method/ (122383)

81 information processing/ or "decision tree"/ or system analysis/ or thematic analysis/ (213613)

82 exp medical record/ (166024)

83 ambulatory care/ or ambulatory care nursing/ or ambulatory monitoring/ (40593)

84 register/ (85768)

85 health survey/ or health status indicator/ (167478)

86 health care quality/ or lot quality assurance sampling/ (203375)

87 exp interview/ (183657)

88 vital statistics/ (5115)

89 exp computer network/ (13561)

90 integrated health care system/ (8191)

91 ((medical or clinical or nursing or dental or public health or community health or rural health or electronic or computer*) adj3 (data or informati* or record or records or network* or registr*)).ti,ab. (399427)

92 ((health or management or medical or clinical) adj3 (system or systems or information)).ti,ab. (220074)

93 or/77-92 (1562200)

94 exp hospital/ (837603)

95 tertiary health care/ or tertiary care center/ (45181)

96 (hospital* or tertiary).ti,ab. (1401752)

97 "review"/ or review.pt. (2178715)

98 "systematic review"/ (103928)

99 (literature review or systematic review).ti. (87119)

100 meta analysis/ (106116)

101 (meta analy* or meta-analy*).ti. (64644)

102 editorial.pt. (500157)

103 cochrane database of systematic reviews.jn. (3772)

104 nonhuman/ (4719785)

105 or/94-104 (8666624)

106 23 and 33 and 51 and 93 (2883)

107 106 not 105 (1632)

108 23 and 33 and 76 and 93 (1929)

109 108 not 105 (1107)

110 limit 107 to exclude medline journals (211)

111 limit 109 to exclude medline journals (83)

1. **CENTRAL Trials Register (Cochrane Library)**

#1 MeSH descriptor: [Community Health Services] explode all trees

12066

#2 MeSH descriptor: [Primary Health Care] this term only

3664

#3 MeSH descriptor: [General Practice] this term only

303

#4 MeSH descriptor: [Community Mental Health Services] this term only

705

#5 MeSH descriptor: [Family Practice] this term only

2176

#6 MeSH descriptor: [Home Care Services] explode all trees

2440

#7 MeSH descriptor: [Physicians, Family] this term only

475

#8 MeSH descriptor: [Community Pharmacy Services] explode all trees

247

#9 MeSH descriptor: [Preventive Health Services] this term only

557

#10 MeSH descriptor: [Community Health Workers] this term only

290

#11 MeSH descriptor: [Allied Health Personnel] this term only

208

#12 MeSH descriptor: [Midwifery] this term only

293

#13 MeSH descriptor: [Volunteers] this term only

211

#14 MeSH descriptor: [Community Health Nursing] this term only

381

#15 ("primary care" or "primary health care" or "primary healthcare" or "primary medical care" or "community health" or "rural health" or "district health"):ti,ab

11280

#16 ((community or family or general) near/2 (practice or practitioner* or physician* or doctor*)):ti,ab

7398

#17 (community near/2 ("mental health" or psychiatr* or care or pharmac*)):ti,ab

1695

#18 "preventive health service*":ti,ab

23

#19 ((("community health" or "village health") near/3 (aide* or worker*)) or "barefoot doctor*"):ti,ab

410

#20 (allied health personnel or allied health worker* or paramedical personnel or paramedics):ti,ab

331

#21 ("birth attendant*" or midwif* or mid-wif* or midwiv* or mid-wiv* or "traditional birth" or doula*):ti,ab

914

#22 ("voluntary worker*" or "volunteer worker*"):ti,ab

2

#23 (lay near ("health work*" or "health personnel")):ti,ab

61

#24 {or #1-#23}

30579

#25 MeSH descriptor: [Developing Countries] this term only

948

#26 ("less-developed countr*" or "third world countr*" or "under developed countr*" or "underdeveloped countr*" or "developing nation*" or "less developed nation*" or "third world nation*" or "under developed nation*" or "underdeveloped nation*" or "developing countr*"):ti,ab

1949

#27 ("low income count*" or "low income nation*" or "middle income count*" or "middle income nation*" or "low and middle income" or lmic or lmics):ti,ab

696

#28 [mh Africa]

5222

#29 MeSH descriptor: [Asia] explode all trees

13971

#30 MeSH descriptor: [Caribbean Region] explode all trees

346

#31 [mh "central america"] or [mh "south america"] or [mh "latin america"] or [mh mexico]

2603

#32 MeSH descriptor: [Indian Ocean Islands] explode all trees

125

#33 MeSH descriptor: [Melanesia] explode all trees

66

#34 MeSH descriptor: [Micronesia] explode all trees

4

#35 MeSH descriptor: [Polynesia] explode all trees

115

#36 MeSH descriptor: [Pacific Islands] this term only

17

#37 MeSH descriptor: [Oceania] this term only

2

#38 {or #25-#37}

23806

#39 MeSH descriptor: [Epidemiologic Studies] this term only

60

#40 MeSH descriptor: [Case-Control Studies] explode all trees

13175

#41 MeSH descriptor: [Cohort Studies] explode all trees

126183

#42 (cohort near (study or studies)):ti,ab

6362

#43 "Cohort analy*":ti,ab

187

#44 ("Follow up" near (study or studies)):ti,ab

9700

#45 MeSH descriptor: [Observational Study] this term only

0

#46 MeSH descriptor: [Observational Studies as Topic] explode all trees

44

#47 (observational near (study or studies)):ti,ab

5289

#48 (longitudinal or retrospective or "cross sectional"):ti,ab

19166

#49 MeSH descriptor: [Cross-Sectional Studies] this term only

3801

#50 ((Ecologic* or Panel) near (study or studies)):ti,ab

245

#51 {or #39-#50}

155520

#52 "randomized controlled trial":pt

395450

#53 "controlled clinical trial":pt

88155

#54 (randomly or randomi?ed or placebo or trial or groups):ab

534744

#55 ((before near/5 after) or (pre near/5 post) or pretest or "pre test" or posttest or "post test" or quasiexperiment* or "quasi experiment*" or "time series" or "time point*"):ti,ab

70694

#56 {or #52-#55}

660276

#57 MeSH descriptor: [Data Collection] this term only

1293

#58 MeSH descriptor: [Data Accuracy] this term only

1

#59 MeSH descriptor: [Datasets as Topic] this term only

4

#60 MeSH descriptor: [Focus Groups] this term only

382

#61 MeSH descriptor: [Health Impact Assessment] this term only

9

#62 MeSH descriptor: [Interviews as Topic] this term only

1494

#63 MeSH descriptor: [Lot Quality Assurance Sampling] this term only

0

#64 MeSH descriptor: [Narration] this term only

130

#65 MeSH descriptor: [Records as Topic] this term only

34

#66 MeSH descriptor: [Registries] this term only

835

#67 MeSH descriptor: [Surveys and Questionnaires] this term only

19761

#68 MeSH descriptor: [Vital Statistics] 5 tree(s) exploded

30510

#69 MeSH descriptor: [Medical Informatics] this term only

66

#70 MeSH descriptor: [Health Information Exchange] this term only

3

#71 MeSH descriptor: [Nursing Informatics] this term only

9

#72 MeSH descriptor: [Public Health Informatics] this term only

5

#73 MeSH descriptor: [Dental Informatics] this term only

2

#74 MeSH descriptor: [Medical Informatics Applications] this term only

24

#75 MeSH descriptor: [Medical Records] this term only

729

#76 MeSH descriptor: [Medical Record Linkage] this term only

29

#77 MeSH descriptor: [Medical Records Systems, Computerized] this term only

239

#78 MeSH descriptor: [Electronic Health Records] this term only

188

#79 MeSH descriptor: [Nursing Records] this term only

42

#80 MeSH descriptor: [Ambulatory Care Information Systems] this term only

27

#81 MeSH descriptor: [Medical Order Entry Systems] this term only

75

#82 MeSH descriptor: [Decision Support Systems, Management] this term only

8

#83 MeSH descriptor: [Registries] explode all trees

879

#84 MeSH descriptor: [Information Management] explode all trees

30

#85 MeSH descriptor: [Computer Communication Networks] this term only

73

#86 ((medical or clinical or nursing or dental or "public health" or "community health" or "rural health" or electronic or computer*) near/3 (data or informati* or record or records or network* or registr*)):ti,ab

17588

#87 MeSH descriptor: [Health Information Management] this term only

9

#88 MeSH descriptor: [Systems Analysis] this term only

20

#89 MeSH descriptor: [Systems Integration] this term only

29

#90 MeSH descriptor: [Health Information Systems] this term only

8

#91 MeSH descriptor: [Management Information Systems] this term only

12

#92 MeSH descriptor: [Personnel Staffing and Scheduling Information Systems] this term only

1

#93 ((health or management or medical or clinical) near/3 (system or systems or information)):ti,ab

6545

#94 {or #57-#93}

72288

#95 MeSH descriptor: [Hospitals] explode all trees

3403

#96 MeSH descriptor: [Tertiary Healthcare] this term only

6

#97 MeSH descriptor: [Tertiary Care Centers] this term only

108

#98 (hospital* or tertiary):ti,ab

72140

#99 {or #95-#98}

73362

#100 #24 and #38 and #51 and #94

197

#101 #100 and not #99

158 in Trials

#102 #24 and #38 and #56 and #94

699

#103 #102 and not #99

574 in Trials

1. **WHO Global Health Library – Regional Offices**
2. RCTs Search

(("primary care" or "primary health care" or "primary healthcare" or "primary medical care" or "community health" or "rural health" or "district health") OR ((community or family or general) AND (practice or practitioner* or physician* or doctor*)) or (community AND ("mental health" or psychiatr* or care or pharmac*)) OR ((("community health" or "village health") AND (aide* or worker*)) or "barefoot doctor*") OR ("allied health personnel" or "allied health worker*" or "paramedical personnel" or paramedics) OR ("birth attendant*" or midwif* or mid-wif* or midwiv* or mid-wiv* or "traditional birth" or doula*) OR ("voluntary worker*" or "volunteer worker*") OR (lay AND ("health work*" or "health personnel")) OR MH:("Community Health Services" OR "Primary Health Care" OR "General practice" OR "Community mental health services" OR "Family practice" OR "Home care services" OR "Family physicians" OR "Community pharmacy services" OR "Preventive health services" OR "Community Health Aides" OR "Allied Health Personnel" OR Midwifery OR "Voluntary Workers" OR "Community Health Nursing")) AND (((medical or clinical or nursing or dental or "public health" or "community health" or "rural health" or electronic or computer*) AND (data or informati* or record or records or network* or registr*)) or ((health or management or medical or clinical) AND (system or systems or information)) OR MH:("data collection" or "data accuracy" or "datasets as topic" or "focus groups" or "health impact assessment" or "interviews as topic" or "lot quality assurance sampling" or narration or "records as topic" or registries or "surveys and questionnaires" or "vital statistics" OR "medical informatics" OR "health information exchange" OR "nursing informatics" OR "public health informatics" OR "dental informatics" OR "medical informatics applications" OR "medical records" OR "medical record linkage" OR "medical record systems, computerized" OR "electronic health records" OR "nursing records" OR "ambulatory care information systems" OR "medical order entry system" OR "decision support systems, management" OR registries OR "information management" OR "computer communication networks" OR "health information management" OR "systems analysis" OR "systems integration" OR "health information systems" OR "management information systems" OR "personnel staffing and scheduling information systems")) AND (PT:"randomized controlled trial" OR PT:"controlled clinical trial" OR PT:"multicenter study" OR MH:"randomized controlled trials as topic" OR MH:"controlled clinical trials as topic" OR MH:"multicenter studies as topic" OR MH:"random allocation" OR MH:"double-blind method" OR MH:"single-blind method" OR ((TW:before AND after) or (TW:pre AND post) or TW:pretest or TW:"pre test" or TW:posttest or TW:"post test" or TW:quasiexperiment* or TW:"quasi experiment*" or TW:"time series" or TW:"time point*") OR ((ensaio$ OR ensayo$ OR trial$) AND (azar OR acaso OR placebo OR control$ OR aleat$ OR random$ OR enmascarado$ OR simpleciego OR ((simple$ OR single OR duplo$ OR doble$ OR double$) AND (cego OR ciego OR blind OR mask))) AND clinic$)) AND NOT (MH:animals OR MH:rabbits OR MH:rats OR MH:primates OR MH:dogs OR MH:cats OR MH:swine OR PT:"in vitro") **– 591 Hits (Medline refs excluded)**

1. Observational Studies Search

TW:(("primary care" or "primary health care" or "primary healthcare" or "primary medical care" or "community health" or "rural health" or "district health") OR ((community or family or general) AND (practice or practitioner* or physician* or doctor*)) or TW:(community AND ("mental health" or psychiatr* or care or pharmac*)) OR TW:((("community health" or "village health") AND (aide* or worker*)) or "barefoot doctor*") OR ("allied health personnel" or "allied health worker*" or "paramedical personnel" or paramedics) OR ("birth attendant*" or midwif* or mid-wif* or midwiv* or mid-wiv* or "traditional birth" or doula*) OR ("voluntary worker*" or "volunteer worker*") OR (lay AND ("health work*" or "health personnel")) OR MH:("Community Health Services" OR "Primary Health Care" OR "General practice" OR "Community mental health services" OR "Family practice" OR "Home care services" OR "Family physicians" OR "Community pharmacy services" OR "Preventive health services" OR "Community Health Aides" OR "Allied Health Personnel" OR Midwifery OR "Voluntary Workers" OR "Community Health Nursing")) AND (((medical or clinical or nursing or dental or "public health" or "community health" or "rural health" or electronic or computer*) AND (data or informati* or record or records or network* or registr*)) or ((health or management or medical or clinical) AND (system or systems or information)) OR MH:("data collection" or "data accuracy" or "datasets as topic" or "focus groups" or "health impact assessment" or "interviews as topic" or "lot quality assurance sampling" or narration or "records as topic" or registries or "surveys and questionnaires" or "vital statistics" OR "medical informatics" OR "health information exchange" OR "nursing informatics" OR "public health informatics" OR "dental informatics" OR "medical informatics applications" OR "medical records" OR "medical record linkage" OR "medical record systems, computerized" OR "electronic health records" OR "nursing records" OR "ambulatory care information systems" OR "medical order entry system" OR "decision support systems, management" OR registries OR "information management" OR "computer communication networks" OR "health information management" OR "systems analysis" OR "systems integration" OR "health information systems" OR "management information systems" OR "personnel staffing and scheduling information systems")) AND MJ:("epidemiologic studies" OR "case control studies" OR "retrospective studies" OR "cohort studies" OR "longitudinal studies" OR "follow-up studies" OR "prospective studies" OR "observational study" OR "cross-sectional studies") OR TW:("cohort study" OR "cohort studies" OR "cohort analy*" OR "follow up stud*" OR "observational stud*" OR longitudinal OR retrospective OR cross-sectional OR "cross sectional" OR "ecologic* stud*" OR "panel stud*") AND NOT (MH:(hospitals OR Tertiary Care Centers OR Tertiary Healthcare) OR TW:( hospital* OR tertiary)) **– 3455 hits (with Subject heading filters applied and Medline refs excluded)**

1. **WHO ICTRP**

Advanced Search: Searched by health topic

1. Community health 151

2. Health systems 7

3. Informatics 13

4. Primary health care 356

1. **Health Systems Evidence**

Advanced search – guided – 165 hits

Filters selected: All LMICs; Any system arrangement; Sectors: Primary health, Home care, Public health; Any implementation strategy; Any technology.

1. **Epistemonikos**

1. (title:(primary care OR primary health care OR primary healthcare OR primary medical care OR community health OR rural health OR district health) OR abstract:(primary care OR primary health care OR primary healthcare OR primary medical care OR community health OR rural health OR district health)) AND (title:( data OR informatics OR information OR record OR records OR network OR networks OR networking OR registry OR registries *) OR abstract:( data OR informatics OR information OR record OR records OR network OR networks OR networking OR registry OR registries))

AND LMICs AND RCTs (Primary studies) – 268 hits

2. (title:(primary care OR primary health care OR primary healthcare OR primary medical care OR community health OR rural health OR district health) OR abstract:(primary care OR primary health care OR primary healthcare OR primary medical care OR community health OR rural health OR district health)) AND (title:( data OR informatics OR information OR record OR records OR network OR networks OR networking OR registry OR registries *) OR abstract:( data OR informatics OR information OR record OR records OR network OR networks OR networking OR registry OR registries))

AND LMICs AND non-RCTs (Primary studies) – 189 hits

**8. PDQ – Evidence**

1. (title:(primary care OR primary health care OR primary healthcare OR primary medical care OR community health OR rural health OR district health) OR abstract:(primary care OR primary health care OR primary healthcare OR primary medical care OR community health OR rural health OR district health)) AND (title:( data OR informatics OR information OR record OR records OR network OR networks OR networking OR registry OR registries *) OR abstract:( data OR informatics OR information OR record OR records OR network OR networks OR networking OR registry OR registries))

AND LMICs AND RCTs (Primary studies) – 173 hits

2. (title:(primary care OR primary health care OR primary healthcare OR primary medical care OR community health OR rural health OR district health) OR abstract:(primary care OR primary health care OR primary healthcare OR primary medical care OR community health OR rural health OR district health)) AND (title:( data OR informatics OR information OR record OR records OR network OR networks OR networking OR registry OR registries *) OR abstract:( data OR informatics OR information OR record OR records OR network OR networks OR networking OR registry OR registries))

AND LMICs AND non- RCTs (Primary studies) – 160 hits
