# Supplementary material for: Framework synthesis to inform the ideation and design of a paper‐based health information system (PHISICC)
Source: Int J Health Plann Manage. 2022 Apr 23;37(4):1953–72. doi: 10.1002/hpm.3487 (PMC9544999; doi:10.1002/hpm.3487)
Supplement: Supplementary file 3 — Supplementary Material [file HPM-37-1953-s001.docx]

**Supplementary file 3: List of Included Studies and List of Excluded Studies**

*Article title*: Research on Health Information Systems Focus on Technical Aspects Rather than on Decision-Making. A Framework Synthesis to Inform the Ideation of Paper-based Health Information Systems (PHISICC).

*Journal name*: IJHPM

*Authors information including author names, affiliation, and email address of the corresponding author*:

Meike-Kathrin Zuske^1, 2^, Christian Auer^1, 2^, Sandy Oliver^3,4^, John Eyers^5^, Xavier Bosch-Capblanch^1, 2^ ^*^

^1^ Swiss Tropical and Public Health Institute, Basel, Switzerland;

^2^ University of Basel, Basel, Switzerland

^3^ University College London, EPPI-Centre, Social Research Institute, London, United Kingdom

^4^ University of Johannesburg, Africa Centre for Evidence, Faculty of Humanities, Johannesburg, South Africa

^5^ Independent Consultant & Senior Research Fellow, 3ie, c/o LIDC, 20 Bloomsbury Square, London WC1A 2NS, United Kingdom

^*^ Correspondence: [x.bosch@unibas.ch](mailto:x.bosch@unibas.ch)

1. **List if included studies**
2. Abud SM, G. M. (2015) ' Records of growth and development data in the child health handbook', Rev Gaucha Enferm, 36, pp. 97-105.
3. Al Baho, A. K., El Essa, I. and Al Assousi, M. (2003) 'Perceptions of patients, doctors and clerks of their roles in causing problems in medical records', KMJ - Kuwait Medical Journal, 35(1), pp. 13-17.
4. Alberti, H., Boudriga, N. and Nabli, M. (2006) 'Disease-specific medical records improve the recording of processes of care in the management of type 2 diabetes mellitus', Public Health, 120(7), pp. 650-3.
5. Al-Hashimi, D. A., Al-Roomi, K. and Al-Sayyad, A. S. (2014) 'Reasons for under-reporting of notifiable communicable diseases in the Kingdom of Bahrain: A health-centers based survey', Journal of the Bahrain Medical Society, 25(2), pp. 75-79.
6. Alves, C. R., Lasmar, L. M., Goulart, L. M., Alvim, C. G., Maciel, G. V., Viana, M. R., Colosimo, E. A., Carmo, G. A., Costa, J. G., Magalhaes, M. E., Mendonca, M. L., Beirao, M. M. and Moulin, Z. S. (2009) '[Quality of data on the Child Health Record and related factors]', Cadernos de Saude Publica, 25(3), pp. 583-95.
7. Amoakoh-Coleman, M., Kayode, G. A., Brown-Davies, C., Agyepong, I. A., Grobbee, D. E., Klipstein-Grobusch, K. and Ansah, E. K. (2015) 'Completeness and accuracy of data transfer of routine maternal health services data in the greater Accra region', BMC Research Notes, 8, pp. 114.
8. Azandegbe, N., Testa, J. and Makoutode, M. (2004) '[Assessment of partogram utilisation in Benin]', Sante, 14(4), pp. 250-5.
9. Barboza, C. L., Barreto, M. d. S. and Marcon, S. S. (2012) 'Childcare records in primary care: a descriptive study', Online braz. j. nurs. (Online), 11(2).
10. Bogaerts, J., Vuylsteke, B., Martinez Tello, W., Mukantabana, V., Akingeneye, J., Laga, M. and Piot, P. (1995) 'Simple algorithms for the management of genital ulcers: evaluation in a PHCcentre in Kigali, Rwanda', Bulletin of the World Health Organization, 73(6), pp. 761-7.
11. Broomhead, S. and Mars, M. (2011) 'Retrospective return on investment analysis of an electronic treatment adherence device piloted in the Northern Cape Province', Telemedicine journal and e-health: the official journal of the American Telemedicine Association, 18(1), pp. 24-31.
12. Burke, L., Suswardany, D. L., Michener, K., Mazurki, S., Adair, T., Elmiyati, C. and Rao, C. (2011) 'Utility of local health registers in measuring perinatal mortality: a case study in rural Indonesia', BMC Pregnancy & Childbirth, 11, pp. 20.
13. da Costa, T. M., Salomão, P. L., Martha, A. S., Pisa, I. T. and Sigulem, D. (2009) 'The impact of short message service text messages sent as appointment reminders to patients' cell phones at outpatient clinics in São Paulo, Brazil', International journal of medical informatics, 79(1), pp. 65-70.
14. Dalaba, M. A., Akweongo, P., Williams, J., Saronga, H. P., Tonchev, P., Sauerborn, R., Mensah, N., Blank, A., Kaltschmidt, J. and Loukanova, S. (2014) 'Costs associated with implementation of computer-assisted clinical decision support system for antenatal and delivery care: case study of Kassena-Nankana district of northern Ghana', PLoS ONE [Electronic Resource], 9(9), pp. e106416.
15. DO, S. (2009) ' Towards a sustainable community database: taking advantage of the Road-to-Health cards to monitor and evaluate health interventions targeting under fives', Tanzania J Health Res, 11, pp. 46-50.
16. Doubova, S. V., Lamadrid-Figueroa, H. and Perez-Cuevas, R. (2013) 'Use of electronic health records to evaluate the quality of care for hypertensive patients in Mexican family medicine clinics', Journal of Hypertension, 31(8), pp. 1714-23.
17. Doubova, S. V., Perez-Cuevas, R., Ortiz-Panozo, E. and Hernandez-Prado, B. (2014) 'Evaluation of the quality of antenatal care using electronic health record information in family medicine clinics of Mexico City', BMC Pregnancy & Childbirth, 14, pp. 168.
18. Essen, B., Laurell, L., Pena, R., Ostergren, P. O. and Liljestrand, J. (1994) 'Antenatal cards--what should they contain?', Journal of Tropical Pediatrics, 40(3), pp. 130-2.
19. Galvao, P. R., Ferreira, A. T., Maciel, M. D., De Almeida, R. P., Hinders, D., Schreuder, P. A. and Kerr-Pontes, L. R. (2008) 'An evaluation of the Sinan health information system as used by the Hansen's disease control programme, Pernambuco State, Brazil', Leprosy Review, 79(2), pp. 171-82.
20. Holanda, A. A., do Carmo, E. S. H. L., Vieira, A. P. and Catrib, A. M. (2012) 'Use and satisfaction with electronic health record by primary care physicians in a health district in Brazil', Journal of Medical Systems, 36(5), pp. 3141-9.
21. Jimoh, L., Pate, M. A., Lin, L. and Schulman, K. A. (2012) 'A model for the adoption of ICT by health workers in Africa', International Journal of Medical Informatics, 81(11), pp. 773-81.
22. Joubert, K. and Casoojee, A. (2013) 'Hearing-screening record-keeping practices at primary healthcare clinics in Gauteng', South African Journal of Communication Disorders - die Suid-Afrikaanse Tydskrif vir Kommunikasieafwykings, 60, pp. 27-30.
23. Kijsanayotin, B., Pannarunothai, S. and Speedie, S. (2007) 'Penetration and adoption of health information technology (IT) in Thailand's community health centers (CHCs): a national survey', Studies in Health Technology & Informatics, 129(Pt 2), pp. 1154-8.
24. Kijsanayotin, B., Pannarunothai, S. and Speedie, S. M. (2009) 'Factors influencing health information technology adoption in Thailand's community health centers: applying the UTAUT model', International Journal of Medical Informatics, 78(6), pp. 404-16.
25. Kunimitsu, A. (2009) 'The accuracy of clinical malaria case reporting at PHCfacilities in Honiara, Solomon Islands', Malaria Journal, 8, pp. 80.
26. Leon, N., Surender, R., Bobrow, K., Muller, J. and Farmer, A. (2015) 'Improving treatment adherence for blood pressure lowering via mobile phone SMS-messages in South Africa: a qualitative evaluation of the SMS-text Adherence SuppoRt (StAR) trial', BMC Family Practice, 16, pp. 80.
27. Lima, R. T. d., Costa, G. M. C., Franca, I. S. X. d., Sousa, F. S. d. and Coura, A. S. 'Information system as tool for planning and assessment of health services: descriptive study - O sistema de informacao como ferramenta para planejamento e avaliacao dos servicos de saude: estudo descritivo', Online braz. j. nurs. (Online), 9(2).
28. Lungo, J. H. (2008) 'The Reliability and Usability of District Health Information Software: Case Studies from Tanzania', Tanzania Journal of Health Research, 10(1), pp. 39-45, tab.
29. Ly, B. A., Gagnon, M. P., Legare, F., Rousseau, M. and Simonyan, D. (2015) 'Determinants of Physicians' Intention to Collect Data Exhaustively in Registries: an Exploratory Study in Bamako's Community Health Centres', Ghana Medical Journal, 49(2), pp. 90-6.
30. Mahmood, M. A. and Saniotis, A. (2011) 'Use of syndromic management algorithm for sexually transmitted infections and reproductive tract infections management in community settings in Karachi', JPMA - Journal of the Pakistan Medical Association, 61(5), pp. 453-7.
31. Mahmood, S. and Ayub, M. (2010) 'Accuracy of PHCstatistics reported by community based lady health workers in district Lahore', JPMA - Journal of the Pakistan Medical Association, 60(8), pp. 649-53.
32. Margalit, R. S., Roter, D., Dunevant, M. A., Larson, S. and Reis, S. (2006) 'Electronic medical record use and physician-patient communication: an observational study of Israeli primary care encounters', Patient Education & Counseling, 61(1), pp. 134-41.
33. Mash, B., Powell, D., du Plessis, F., van Vuuren, U., Michalowska, M. and Levitt, N. (2007) 'Screening for diabetic retinopathy in primary care with a mobile fundal camera--evaluation of a South African pilot project', South African Medical Journal. Suid-Afrikaanse Tydskrif Vir Geneeskunde, 97(12), pp. 1284-8.
34. Mghamba, J. and Mboera, L. (2004) 'Challenges of implementing an Integrated Disease Surveillance and Response strategy using the current Health Management Information System in Tanzania', Tanzania Health research Bulletin, 6(2), pp. 57-63.
35. Moimaz, S. A., Garbin, C. A., Garbin, A. J., Zina, L. G., Yarid, S. D. and Francisco, K. M. (2010) '[Prenatal Information System: critical analysis of register in a municipality of Sao Paulo State]', Revista Brasileira de Enfermagem, 63(3), pp. 385-90.
36. Odhiambo-Otieno, G. W. (2005) 'Evaluation criteria for district health management information systems: lessons from the Ministry of Health, Kenya', International Journal of Medical Informatics, 74(1), pp. 31-8.
37. Ogwang, S., Karyabakabo, Z. and Rutebemberwa, E. (2009) 'Assessment of partogram use during labour in Rujumbura Health Sub District, Rukungiri District, Uganda', African Health Sciences, 9 Suppl 1, pp. S27-34.
38. Palombo CNT, D. L., Fujimori E, Toriyama ATM (2014) ' Use and records of child health handbook focused on growth and development', Rev Esc Enferm USP, 48, pp. 59-66.
39. Parham, G. P., Mwanahamuntu, M. H., Pfaendler, K. S., Sahasrabuddhe, V. V., Myung, D., Mkumba, G., Kapambwe, S., Mwanza, B., Chibwesha, C., Hicks, M. L. and Stringer, J. S. (2010) 'eC3--a modern telecommunications matrix for cervical cancer prevention in Zambia', Journal of lower genital tract disease, 14(3), pp. 167-73.
40. Press, Y., Hazzan, R., Clarfield, A. M. and Dwolatzky, T. (2009) 'A semi-structured computerized screening interview for the assessment of older patients in the primary care setting', International Journal on Disability and Human Development, 8(3), pp. 259-266.
41. Queiroga, R. M. d., Andrade, A. d. N., Abrantes, K. S. M. d., Costa, T. S., Sobreira, M. V. and Casimiro, G. S. (2011) 'Aplicabilidade do Sistema de Informação da Atenção Básica no cotidiano de enfermeiros - Applicability of the primary care information system in nurses’ daily lives', Rev. RENE, 12(n.esp), pp. 943-950.
42. Raeisi, A. R., Saghaeiannejad, S., Karimi, S., Ehteshami, A. and Kasaei, M. (2013) 'District health information system assessment: A case study in Iran', Acta Informatica Medica, 21(1), pp. 30-35.
43. Shachak, A., Hadas-Dayagi, M., Ziv, A. and Reis, S. (2009) 'Primary care physicians' use of an electronic medical record system: a cognitive task analysis', Journal of General Internal Medicine, 24(3), pp. 341-8.
44. dos Santos Ribeiro Silva, M. C., Moules, N. J., Silva, L. and Bousso, R. S. 'The 15-minute family interview: a family health strategy tool - Entrevista de 15 minutos: uma ferramenta de abordagem a familia na estrategia saude da familia - Entrevista de 15 minutos: una herramienta de abordaje familiar en la estrategia salud de la familia', Rev Esc Enferm USP, 47(3), pp. 634-639.
45. Sriha Belguith, A., Elmhamdi, S., Bouanene, I., Ben Saad, M., Ben Salah, A., Harizi, C. and Soltani Essoussi, M. (2015) '[Quality of the medical record notification in primary health care]', Tunisie Medicale, 93(3), pp. 148-52.
46. Tan, W. S., Phang, J. S. and Tan, L. K. (2009) 'Evaluating user satisfaction with an electronic prescription system in a primary care group', Annals of the Academy of Medicine, Singapore, 38(6), pp. 494-7.
47. Tarwa C, d. V. F. (2007) ' The use of the Road to Health Card in monitoring child health', SA Fam Pract, 49, pp. 15.
48. Tierney, W. M., Rotich, J. K., Hannan, T. J., Siika, A. M., Biondich, P. G., Mamlin, B. W., Nyandiko, W. M., Kimaiyo, S., Wools-Kaloustian, K., Sidle, J. E., Simiyu, C., Kigotho, E., Musick, B., Mamlin, J. J. and Einterz, R. M. (2007) 'The AMPATH medical record system: creating, implementing, and sustaining an electronic medical record system to support HIV/AIDS care in western Kenya', Studies in health technology and informatics, 129(Pt 1), pp. 372-6.
49. Tseng, C. W., Brook, R. H., Alexander, G. C., Hixon, A. L., Keeler, E. B., Mangione, C. M., Chen, R., Jackson, E. A. and Dudley, R. A. (2010) 'Health information technology and physicians' knowledge of drug costs', American Journal of Managed Care, 16(4), pp. e105-10.
50. Vasconcellos, M. M., Gribel, E. B. and Moraes, I. H. (2008) '[Health records: evaluation of patient health charts in primary care, Rio de Janeiro, Brazil]', Cadernos de Saude Publica, 24 Suppl 1, pp. S173-82.
51. Wakgari, N., Amano, A., Berta, M. and Tessema, G. A. (2015) 'Partograph utilization and associated factors among obstetric care providers in North Shoa Zone, Central Ethiopia: a cross sectional study', African Health Sciences, 15(2), pp. 552-9.
52. **List if excluded studies**
53. Abe, I. M., Goulart, A. C., Santos Junior, W. R., Lotufo, P. A. and Bensenor, I. M. (2010) 'Validation of a stroke symptom questionnaire for epidemiological surveys', Sao Paulo Medical Journal = Revista Paulista de Medicina, 128(4), pp. 225-31.
54. Abraham, S., Joshi, S., Kumar, V., Patwary, A., Pratinidhi, A., Saxena, V. B., Maitra, K., Singh, K. K., Saxena, N. C. and Saxena, B. N. (1991) 'Indian experience of home based mothers card: ICMR task force study', Indian Journal of Pediatrics, 58(6), pp. 795-804.
55. Adi, A. E., Abdu, T., Khan, A., Rashid, M. H., Ebri, U. E., Cockcroft, A. and Andersson, N. (2015) 'Understanding whose births get registered: a cross sectional study in Bauchi and Cross River states, Nigeria', BMC Research Notes, 8, pp. 79.
56. Alamo, S. T., Wagner, G. J., Sunday, P., Wanyenze, R. K., Ouma, J., Kamya, M., Colebunders, R. and Wabwire-Mangen, F. (2012) 'Electronic medical records and same day patient tracing improves clinic efficiency and adherence to appointments in a community based HIV/AIDS care program, in Uganda', AIDS & Behavior, 16(2), pp. 368-74.
57. Allen, C., Jazayeri, D., Miranda, J., Biondich, P. G., Mamlin, B. W., Wolfe, B. A., Seebregts, C., Lesh, N., Tierney, W. M. and Fraser, H. S. (2007) 'Experience in implementing the OpenMRS medical record system to support HIV treatment in Rwanda', Studies in health technology and informatics, 129(Pt 1), pp. 382-6.
58. Allen, C., Manyika, P., Jazayeri, D., Rich, M., Lesh, N. and Fraser, H. (2006) 'Rapid deployment of electronic medical records for ARV rollout in rural Rwanda', AMIA. Annual Symposium proceedings / AMIA Symposium. AMIA Symposium, pp. 840.
59. Amoroso, C. L., Akimana, B., Wise, B. and Fraser, H. S. (2010) 'Using electronic medical records for HIV care in rural Rwanda', Studies in health technology and informatics, 160(Pt 1), pp. 337-41.
60. Anonymous (1999) 'Visual inspection with acetic acid for cervical-cancer screening: test qualities in a primary-care setting. University of Zimbabwe/JHPIEGO Cervical Cancer Project', Lancet, 353(9156), pp. 869-73.
61. Arvind, C., Manjit, S., Deepak, S. and Anuradha, V. (2012) 'CRD Pune (India) rheumatology/RA database (db): A decade of 'numbers game' and awakening', Indian Journal of Rheumatology, 7, pp. S20-S21.
62. Aytekin, N. T., Pala, K., Akis, N., Nacarkucuk, S. and Aytekin, H. (2002) 'The comparison of reproductive health data in a rural district in Turkey (1981-2001)', European Journal of Contraception & Reproductive Health Care, 7(4), pp. 234-7.
63. Beltrán, F. and Banuelos, A. (1994) 'Epidemiologia y sistemas de información - Epidemiology and information systems', pp. 110-112.
64. Ben, A. J., Neumann, C. R. and Mengue, S. S. (2012) 'The Brief Medication Questionnaire and Morisky-Green test to evaluate medication adherence', Revista de Saude Publica, 46(2), pp. 279-89.
65. Bentur, N. and King, Y. (2010) 'The challenge of validating SF-12 for its use with community-dwelling elderly in Israel', Quality of Life Research, 19(1), pp. 91-5.
66. Blaya, J. and Fraser, H. S. (2006) 'Development, implementation and preliminary study of a PDA-based tuberculosis result collection system', AMIA. Annual Symposium proceedings / AMIA Symposium. AMIA Symposium, pp. 41-5.
67. Breuer, E., Stoloff, K., Myer, L., Seedat, S., Stein, D. J. and Joska, J. (2012) 'Reliability of the lay adherence counsellor administered substance abuse and mental illness symptoms screener (SAMISS) and the International HIV Dementia Scale (IHDS) in a primary care HIV clinic in Cape Town, South Africa', AIDS & Behavior, 16(6), pp. 1464-71.
68. Bricks, L. F., Pannuti, C. S., Sato, H. K., Vico, E. S., de Faria, A. M., Souza, V. V., Sumita, L. M., Costa Ide, C. and Baldacci, E. R. (2007) 'Reliability of information on varicella history in preschool children', Clinics (Sao Paulo, Brazil), 62(3), pp. 309-14.
69. Bussmann, H., Wester, C. W., Ndwapi, N., Vanderwarker, C., Gaolathe, T., Tirelo, G., Avalos, A., Moffat, H. and Marlink, R. G. (2006) 'Hybrid data capture for monitoring patients on highly active antiretroviral therapy (HAART) in urban Botswana', Bulletin of the World Health Organization, 84(2), pp. 127-31.
70. Byass, P., Berhane, Y., Emmelin, A., Kebede, D., Andersson, T., Hogberg, U. and Wall, S. (2002) 'The role of demographic surveillance systems (DSS) in assessing the health of communities: an example from rural Ethiopia', Public Health, 116(3), pp. 145-50.
71. Camozzato, A. L., Godinho, C., Kochhann, R., Massochini, G. and Chaves, M. L. (2015) 'Validity of the Brazilian version of the Neuropsychiatric Inventory Questionnaire (NPI-Q)', Arquivos de Neuro-Psiquiatria, 73(1), pp. 41-5.
72. Castelo, M. S., Coelho-Filho, J. M., Carvalho, A. F., Lima, J. W., Noleto, J. C., Ribeiro, K. G. and Siqueira-Neto, J. I. (2010) 'Validity of the Brazilian version of the Geriatric Depression Scale (GDS) among primary care patients', International Psychogeriatrics, 22(1), pp. 109-13.
73. Chile. Ministry of, H. (1995) 'Visiting card: Health situation in Chile 1995', pp. 29-29.
74. Clevenbergh, P., Van der Borght, S. F., van Cranenburgh, K., Janssens, V., Kitenge Lubangi, C., Gahimbaza, L., Lange, J. M., Rinke de Wit, T. F. and Rijckborst, H. (2006) 'Database-supported teleconferencing: an additional clinical mentoring tool to assist a multinational company HIV/AIDS treatment program in Africa', HIV Clinical Trials, 7(5), pp. 255-62.
75. Curioso, W. H. and Kurth, A. E. (2007) 'Access, use and perceptions regarding Internet, cell phones and PDAs as a means for health promotion for people living with HIV in Peru', BMC medical informatics and decision making, 7, pp. 24.
76. De Souza, N. C., Botelho, C. A. and Honer, M. R. (2004) 'Retrospective study of a pioneer antenatal screening program with 8,477 pregnant women in Brazil', Clinical & Experimental Obstetrics & Gynecology, 31(3), pp. 217-20.
77. Derakhshan, A., Khadem, N., Tabatabaei, M. K. and Mazlouman, S. J. (2003) 'Pain tracker diagnostic instrument: effect on patients&#039; satisfaction with their interactions with the primary care physician', East Mediterr Health J, 9(1-2), pp. 113-122.
78. Diero, L., Rotich, J. K., Bii, J., Mamlin, B. W., Einterz, R. M., Kalamai, I. Z. and Tierney, W. M. (2006) 'A computer-based medical record system and personal digital assistants to assess and follow patients with respiratory tract infections visiting a rural Kenyan health centre', BMC Medical Informatics & Decision Making, 6, pp. 21.
79. Eimeri, T. S. and White, K. L. (1960) 'Organized curiosity: A practical approach to the problem of keeping records for research purposes in general practice', PAHO. Scientific Públication, (534), pp. 186-189.
80. Eisele, T. P., Silumbe, K., Yukich, J., Hamainza, B., Keating, J., Bennett, A. and Miller, J. M. (2013) 'Measuring coverage in MNCH: accuracy of measuring diagnosis and treatment of childhood malaria from household surveys in Zambia', PLoS Medicine / Public Library of Science, 10(5), pp. e1001417.
81. Farley, J., Miller, E., Zamani, A., Tepper, V., Morris, C., Oyegunle, M., Lin, M., Charurat, M. and Blattner, W. (2010) 'Screening for hazardous alcohol use and depressive symptomatology among HIV-infected patients in Nigeria: Prevalence, predictors, and association with adherence', Journal of the International Association of Physicians in AIDS Care, 9(4), pp. 218-226.
82. Franco-Marina, F., Fernandez-Plata, R., Torre-Bouscoulet, L., Garcia-Sancho, C., Sanchez-Gallen, E., Martinez, D., Perez-Padilla, R. and Study, T. (2014) 'Efficient screening for COPD using three steps: a cross-sectional study in Mexico City', NPJ Primary Care Respiratory Medicine, 24, pp. 14002.
83. Fraser, H. S., Jazayeri, D., Nevil, P., Karacaoglu, Y., Farmer, P. E., Lyon, E., Fawzi, M. K., Leandre, F., Choi, S. S. and Mukherjee, J. S. (2004) 'An information system and medical record to support HIV treatment in rural Haiti', BMJ (Clinical research ed.), 329(7475), pp. 1142-6.
84. Giveon, S., Yaphe, J., Hekselman, I., Mahamid, S. and Hermoni, D. (2009) 'The e-patient: a survey of israeli primary care physicians' responses to patients' use of online information during the consultation', Israel Medical Association Journal: Imaj, 11(9), pp. 537-41.
85. Goycochea-Robles, M. V., Sanin, L. H., Moreno-Montoya, J., Alvarez-Nemegyei, J., Burgos-Vargas, R., Garza-Elizondo, M., Rodriguez-Amado, J., Madariaga, M. A., Zamudio, J. A., Cuervo, G. E., Cardiel-Rios, M. H., Pelaez-Ballestas, I. and Grupo de Estudio Epidemiologico de Enfermedades Musculo, A. (2011) 'Validity of the COPCORD core questionnaire as a classification tool for rheumatic diseases', Journal of Rheumatology - Supplement, 86, pp. 31-5.
86. Haberer, J. E., Kiwanuka, J., Nansera, D., Wilson, I. B. and Bangsberg, D. R. (2010) 'Challenges in using mobile phones for collection of antiretroviral therapy adherence data in a resource-limited setting', AIDS and behavior, 14(6), pp. 1294-301.
87. Hamainza, B., Killeen, G. F., Kamuliwo, M., Bennett, A. and Yukich, J. O. (2014) 'Comparison of a mobile phone-based malaria reporting system with source participant register data for capturing spatial and temporal trends in epidemiological indicators of malaria transmission collected by community health workers in rural Zambia', Malaria Journal, 13, pp. 489.
88. Hamza, A. A. Y. (1994) 'comparison between the completeness of referral letters from primary to secondary health care centres in Bahrain in 1992 and 1993', JBMS - Journal of the Bahrain Medical Society, 6(1), pp. 14-16.
89. Harzheim, E., Starfield, B., Rajmil, L., Alvarez-Dardet, C. and Stein, A. T. (2006) '[Internal consistency and reliability of Primary Care Assessment Tool (PCATool-Brasil) for child health services]', Cadernos de Saude Publica, 22(8), pp. 1649-59.
90. Heng, B. H., Sun, Y., Cheah, J. T. S. and Jong, M. (2010) 'The Singapore National Healthcare Group Diabetes Registry--descriptive epidemiology of type 2 diabetes mellitus', Ann Acad Med Singapore, 39(5), pp. 348-52.
91. Hermida, J., Broughton, E. I. and Miller Franco, L. (2011) 'Validity of self-assessment in a quality improvement collaborative in Ecuador', International Journal for Quality in Health Care, 23(6), pp. 690-6.
92. Hewett, P. C., Haberland, N., Apicella, L. and Mensch, B. S. (2012) 'The (mis)reporting of male circumcision status among men and women in Zambia and Swaziland: a randomized evaluation of interview methods', PLoS ONE [Electronic Resource], 7(5), pp. e36250.
93. Heydari, G., Jianfar, G., Alvanpour, A., Hesami, Z., Talischi, F. and Masjedi, M. R. (2011) 'Efficacy of telephone quit-line for smokers in iran: 12 months follow up results', Tanaffos, 10(3), pp. 42-48.
94. Jafarey, S. N., Rizvi, T., Koblinsky, M. and Kureshy, N. (2009) 'Verbal autopsy of maternal deaths in two districts of Pakistan--filling information gaps', Journal of Health, Population & Nutrition, 27(2), pp. 170-83.
95. Jain, A. K., Sathar, Z., Salim, M. and Shah, Z. H. (2013) 'The importance of public sector health facility-level data for monitoring changes in maternal mortality risks among communities: the case of pakistan', Journal of Biosocial Science, 45(5), pp. 601-13.
96. Jazayeri, D., Farmer, P., Nevil, P., Mukherjee, J. S., Leandre, F. and Fraser, H. S. (2003) 'An Electronic Medical Record system to support HIV treatment in rural Haiti', AMIA. Annual Symposium proceedings / AMIA Symposium. AMIA Symposium, pp. 878.
97. Jiang, L., Lee, V. J., Lim, W. Y., Chen, M. I., Chen, Y., Tan, L., Lin, R. T., Leo, Y. S., Barr, I. and Cook, A. R. (2015) 'Performance of case definitions for influenza surveillance', Euro Surveillance: Bulletin Europeen sur les Maladies Transmissibles = European Communicable Disease Bulletin, 20(22), pp. 21145.
98. Kahan, N. R., Waitman, D. A. and Vardy, D. A. (2009) 'Curtailing laboratory test ordering in a managed care setting through redesign of a computerized order form', American Journal of Managed Care, 15(3), pp. 173-6.
99. Kamadjeu, R. M., Tapang, E. M. and Moluh, R. N. (2005) 'Designing and implementing an electronic health record system in primary care practice in sub-Saharan Africa: a case study from Cameroon', Informatics in Primary Care, 13(3), pp. 179-86.
100. Kara, B., Mukaddes, N. M., Altinkaya, I., Guntepe, D., Gokcay, G. and Ozmen, M. (2014) 'Using the modified checklist for autism in toddlers in a well-child clinic in Turkey: adapting the screening method based on culture and setting', Autism, 18(3), pp. 331-8.
101. Karakis, I., Blumenfeld, M., Yegev, Y., Goldfarb, D., Bolotin, A., Weiler, Z. and Carel, R. (2011) 'A computerized surveillance system for asthma', International Journal of Health Care Quality Assurance, 24(4), pp. 308-13.
102. Khader, A., Ballout, G., Shahin, Y., Hababeh, M., Farajallah, L., Zeidan, W., Abu-Zayed, I., Kochi, A., Harries, A. D., Zachariah, R., Kapur, A., Shaikh, I. and Seita, A. (2014) 'Treatment outcomes in a cohort of Palestine refugees with diabetes mellitus followed through use of E-Health over 3 years in Jordan', Tropical Medicine & International Health, 19(2), pp. 219-23.
103. Khanal, S., Gc, V. S., Dawson, P. and Houston, R. (2011) 'Verbal autopsy to ascertain causes of neonatal deaths in a community setting: a study from Morang, Nepal', Jnma, Journal of the Nepal Medical Association, 50(181), pp. 21-7.
104. Kilic, C., Rezaki, M., Rezaki, B., Kaplan, I., Ozgen, G., Sagduyu, A. and Ozturk, M. O. (1997) 'General Health Questionnaire (GHQ12 & GHQ28): psychometric properties and factor structure of the scales in a Turkish primary care sample', Social Psychiatry & Psychiatric Epidemiology, 32(6), pp. 327-31.
105. Lam, C. L., Lauder, I. J. and Lam, D. T. (1999) 'How does a change in the administration method affect the reliability of the COOP/WONCA Charts? World Organization of National Colleges, Academies and Academic Associations of General Practitioners/Family Physicians', Family practice, 16(2), pp. 184-9 [Clinical Trial; Randomized Controlled Trial; Research Support, Non-U.S. Gov't]. Available at: http://onlinelibrary.wiley.com/o/cochrane/clcentral/articles/573/CN-00164573/frame.html.
106. Lau, Y. K., Cassidy, T., Hacking, D., Brittain, K., Haricharan, H. J. and Heap, M. (2014) 'Antenatal health promotion via short message service at a Midwife Obstetrics Unit in South Africa: A mixed methods study', BMC pregnancy and childbirth, 14(1) [Journal: Article]. DOI: 10.1186/1471-2393-14-284.
107. Lawoyin, T. O. (1994) 'The road to improved registration of vital statistics at community level', West African Journal of Medicine, 13(4), pp. 237-41.
108. Lee, K. F., Chan, M. L. and Jun, L. (2011) 'A pilot follow-up study on the use of a reminder system among patients with unsatisfactory control of diabetes mellitus in a Hong Kong public family medicine clinic', Hong Kong Practitioner, 33(2), pp. 56-62.
109. Leny SS, S. A., Sutan R (2013) ' Usage of home-based maternal health record in antenatal monitoring among Malaysian women attended University Kebangsaan Malaysia Medical Centre', Malaysian J Public Health Med, 13.
110. Lim, P. H. and Lee, C. L. (1990) 'Computer application in a general practice', Ann Acad Med Singapore, 19(5), pp. 736-40.
111. Lotrakul, M., Sumrithe, S. and Saipanish, R. (2008) 'Reliability and validity of the Thai version of the PHQ-9', BMC Psychiatry, 8, pp. 46.
112. Luna, D., Franco, M., Plaza, C., Otero, C., Wassermann, S., Gambarte, M. L., Giunta, D. and Gonzalez Bernaldo de Quiros, F. (2013) 'Accuracy of an electronic problem list from primary care providers and specialists', Studies in Health Technology & Informatics, 192, pp. 417-21.
113. Luo, N., Koh, W. P., Ng, W. Y., Yau, J. W., Lim, L. K., Sim, S. S. and Tay, E. G. (2009) 'Acceptance of information and communication technologies for healthcare delivery: a SingHealth Polyclinics study', Annals of the Academy of Medicine, Singapore, 38(6), pp. 529-8.
114. Lynam, P., Rabinovitz, L. M. and Shobowale, M. (1993) 'Using self-assessment to improve the quality of family planning clinic services', Studies in Family Planning, 24(4), pp. 252-60.
115. Marques Júnior, E. T. A., Maciel Filho, R. and August, P. N. 'Overcoming health inequity: potential benefits of a patient-centered open-source public health infostructure - Superando a falta de eqüidade em saúde: benefícios potenciais de uma estrutura de informação em saúde pública, centrada no paciente e de domínio público', Cad Saude Publica, 24(3), pp. 547-557.
116. Martins, P., Rosado-Pinto, J., do Ceu Teixeira, M., Neuparth, N., Silva, O., Tavares, H., Spencer, J. L., Mascarenhas, D., Papoila, A. L., Khaltaev, N. and Annesi-Maesano, I. (2009) 'Under-report and underdiagnosis of chronic respiratory diseases in an African country', Allergy, 64(7), pp. 1061-7.
117. Mbewe, E. K., Uys, L. R. and Birbeck, G. L. (2013) 'The impact of a short depression and anxiety screening tool in epilepsy care in PHCsettings in Zambia', American Journal of Tropical Medicine & Hygiene, 89(5), pp. 873-4.
118. Mitchell, M., Hedt, B. L., Eshun-Wilson, I., Fraser, H., John, M. A., Menezes, C., Grobusch, M. P., Jackson, J., Taljaard, J. and Lesh, N. (2012) 'Electronic decision protocols for ART patient triaging to expand access to HIV treatment in South Africa: a cross sectional study for development and validation', International Journal of Medical Informatics, 81(3), pp. 166-72.
119. Mohd-Sidik, S., Arroll, B., Goodyear-Smith, F. and Zain, A. M. (2011) 'Screening for depression with a brief questionnaire in a primary care setting: validation of the two questions with help question (Malay version)', International Journal of Psychiatry in Medicine, 41(2), pp. 143-54.
120. Moidu, K., Singh, A. K., Bostrom, K., Wigertz, O., Trell, E. and Kjessler, B. (1992) 'MCHS: an application software for family welfare programmes', Medical Informatics, 17(4), pp. 279-91.
121. Moreno, L., Goldman, N. and Babakol, O. (1991) '[Use of a monthly calendar for collecting retrospective data on contraception: an evaluation of the experimental field studies of the Demographic and Health Surveys (DHS)]', Notas de Poblacion, 18-19(50-52), pp. 11-37.
122. Nahidi, F., Tavafian, S. S., Heidarzadeh, M., Hajizadeh, E. and Montazeri, A. (2014) 'The Mother-Newborn Skin-to-Skin Contact Questionnaire (MSSCQ): development and psychometric evaluation among Iranian midwives', BMC Pregnancy & Childbirth, 14, pp. 85.
123. Nhiwatiwa, S., Patel, V. and Acuda, W. (1998) 'Predicting postnatal mental disorder with a screening questionnaire: a prospective cohort study from Zimbabwe', Journal of Epidemiology & Community Health, 52(4), pp. 262-6.
124. Nur, O. E. (1984) 'The validity of fertility trends from retrospective data: Jordan', Population Bulletin of the United Nations Economic Commission for Western Asia, 24, pp. 127-45.
125. Odigie, V. I., Yusufu, L. M., Dawotola, D. A., Ejagwulu, F., Abur, P., Mai, A., Ukwenya, Y., Garba, E. S., Rotibi, B. B. and Odigie, E. C. (2012) 'The mobile phone as a tool in improving cancer care in Nigeria', Psycho-oncology, 21(3), pp. 332-5.
126. Oluoch, T., Katana, A., Ssempijja, V., Kwaro, D., Langat, P., Kimanga, D., Okeyo, N., Abu-Hanna, A. and de Keizer, N. (2014) 'Electronic medical record systems are associated with appropriate placement of HIV patients on antiretroviral therapy in rural health facilities in Kenya: a retrospective pre-post study', Journal of the American Medical Informatics Association, 21(6), pp. 1009-14.
127. Olweny, C. L. (1985) 'The role of cancer registration in developing countries', IARC Scientific Publications, (66), pp. 143-52.
128. Onono, M. A., Carraher, N., Cohen, R. C., Bukusi, E. A. and Turan, J. M. (2011) 'Use of personal digital assistants for data collection in a multi-site AIDS stigma study in rural south Nyanza, Kenya', African Health Sciences, 11(3), pp. 464-73.
129. Organismo Andino de Salud - Convenio Hipólito, U. and Adecri 2005. Etude complementaire: diagnostic des systemes d´information de la securite sociale des pays de la communaute andine. Dans le cadre de l´application de la composante sanitaire de la Decisión 583. Syntese de l´etude 2005. - Complementary study: diagnosis of the information systems of the social security of the Andean Community countries. Within the framework of the application of the medical component of Decisión 583. Synthesis of the study 2005.
130. Oyama, H., Ono, Y., Watanabe, N., Tanaka, E., Kudoh, S., Sakashita, T., Sakamoto, S., Neichi, K., Satoh, K., Nakamura, K. and Yoshimura, K. (2006) 'Local community intervention through depression screening and group activity for elderly suicide prevention', Psychiatry and clinical neurosciences, 60(1), pp. 110-4 [Controlled Clinical Trial; Multicenter Study]. DOI: 10.1111/j.1440-1819.2006.01468.x.
131. Patel, V., Simunyu, E., Gwanzura, F., Lewis, G. and Mann, A. (1997) 'The Shona Symptom Questionnaire: the development of an indigenous measure of common mental disorders in Harare', Acta Psychiatrica Scandinavica, 95(6), pp. 469-75.
132. Peleg, R., Ostermich, A., Gienco, V. and Portughiez, E. (2013) 'Screening tests among family doctors: do we do as we preach?', Public Health, 127(3), pp. 282-9.
133. Phillips, J. F., Macleod, B. B. and Pence, B. (2000) 'The Household Registration System: computer software for the rapid dissemination of demographic surveillance systems', Demographic Research [electronic resource], 2, pp. [40] p.
134. Prado-Aguilar, C. A., Martinez, Y. V., Segovia-Bernal, Y., Reyes-Martinez, R. and Arias-Ulloa, R. (2009) 'Performance of two questionnaires to measure treatment adherence in patients with Type-2 diabetes', BMC Public Health, 9, pp. 38.
135. Prince, M. J., de Rodriguez, J. L., Noriega, L., Lopez, A., Acosta, D., Albanese, E., Arizaga, R., Copeland, J. R., Dewey, M., Ferri, C. P., Guerra, M., Huang, Y., Jacob, K. S., Krishnamoorthy, E. S., McKeigue, P., Sousa, R., Stewart, R. J., Salas, A., Sosa, A. L., Uwakwa, R. and Dementia research, g. (2008) 'The 10/66 Dementia Research Group's fully operationalised DSM-IV dementia computerized diagnostic algorithm, compared with the 10/66 dementia algorithm and a clinician diagnosis: a population validation study', BMC Public Health, 8, pp. 219.
136. Radhakrishna, S., Satagopan, M. C., Krishnaswami, K. V., Tripathy, S. P. and Fox, W. (1979) 'Efficiency of address cards, experienced health visitors and motivated registry clerks in obtaining the home address of urban patients in South India', Tubercle, 60(3), pp. 150-7.
137. Reis, S., Borkan, J., Vanraalte, R., Tamir, A., Dahan, R., Hermoni, D. and Network, R. A.-I. F. P. R. (2007) 'The LBP patient perception scale: a new predictor of LBP episode outcomes among primary care patients', Patient Education & Counseling, 67(1-2), pp. 191-5.
138. Rocha, N. S. and Fleck, M. P. (2009) 'Validity of the Brazilian version of WHOQOL-BREF in depressed patients using Rasch modelling', Revista de Saude Publica, 43(1), pp. 147-53.
139. Rodrigues, S. C. M. and Damião, G. C. 'Virtual Environment: assistance in nursing care for the deaf based on the protocol of Primary Care - Ambiente virtual: apoyo al servicio de enfermería para los sordos basado en el protocolo de atención básica - Ambiente virtual: auxílio ao atendimento de enfermagem para surdos com base no protocolo de atenção básica', Rev Esc Enferm USP, 48(4), pp. 731-738.
140. Ronsmans, C., Bulut, A., Yolsal, N., Agacfidan, A. and Filippi, V. (1996) 'Clinical algorithms for the screening of Chlamydia trachomatis in Turkish women', Genitourinary Medicine, 72(3), pp. 182-6.
141. Rotheram-Borus, M. J., Tomlinson, M., Gwegwe, M., Comulada, W. S., Kaufman, N. and Keim, M. (2012) 'Diabetes buddies: peer support through a mobile phone buddy system', The Diabetes educator, 38(3), pp. 357-65.
142. Rowe, A. K. (2009) 'Potential of integrated continuous surveys and quality management to support monitoring, evaluation, and the scale-up of health interventions in developing countries', American Journal of Tropical Medicine & Hygiene, 80(6), pp. 971-9.
143. Sánchez-García, S., Juárez-Cedillo, T., García-González, J. J., Espinel-Bermúdez, C., Gallo, J. J., Wagner, F. A., Vázquez-Estupiñán, F. and García-Peña, C. 'Usefulness of two instruments in assessing depression among elderly Mexicans in population studies and for primary care - Utilidad de dos instrumentos para valorar depresión entre adultos mayores de México en estudios poblacionales y para la atención en el primer nivel de salud', Salud Publica Mex, 50(6), pp. 447-456.
144. Scatena, L. M., Wysocki, A. D., Beraldo, A. A., Magnabosco, G. T., Brunello, M. E., Netto Ruffino, A., Nogueira Jde, A., Silva Sobrinho, R. A., Brito, E. W., Alexandre, P. B., Monroe, A. A. and Villa, T. C. (2015) 'Validity and reliability of a health care service evaluation instrument for tuberculosis', Revista de Saude Publica, 49, pp. 7.
145. Shekalaghe, S., Cancino, M., Mavere, C., Juma, O., Mohammed, A., Abdulla, S. and Ferro, S. (2013) 'Clinical performance of an automated reader in interpreting malaria rapid diagnostic tests in Tanzania', Malaria Journal, 12, pp. 141.
146. Siika, A. M., Rotich, J. K., Simiyu, C. J., Kigotho, E. M., Smith, F. E., Sidle, J. E., Wools-Kaloustian, K., Kimaiyo, S. N., Nyandiko, W. M., Hannan, T. J. and Tierney, W. M. (2005) 'An electronic medical record system for ambulatory care of HIV-infected patients in Kenya', International journal of medical informatics, 74(5), pp. 345-55.
147. Simonyan, D., Gagnon, M. P., Duchesne, T. and Roos-Weil, A. (2013) 'Effects of a telehealth programme using mobile data transmission on primary healthcare utilisation among children in Bamako, Mali', Journal of Telemedicine & Telecare, 19(6), pp. 302-6.
148. Singh, A. K., Kohli, M., Trell, E., Wigertz, O. and Kohli, S. (1997) 'Bhorugram (India): revisited. A 4 year follow-up of a computer-based information system for distributed MCH services', International Journal of Medical Informatics, 44(2), pp. 117-25.
149. Sirinavin, S., Suvanakoot, P., Sathapatayavongs, B. and Malatham, K. (1998) 'Effect of antibiotic order form guiding rational use of expensive drugs on cost containment', The Southeast Asian journal of tropical medicine and public health, 29(3), pp. 636-42.
150. Smith, S. and Viviers, J. (2008) 'Electronic data interface in general practice improves debtor days', South African Medical Journal. Suid-Afrikaanse Tydskrif Vir Geneeskunde, 98(4), pp. 238.
151. Stone, K. E., Burrell, L., Higman, S. M., McFarlane, E., Fuddy, L., Sia, C. and Duggan, A. K. (2006) 'Agreement of injury reporting between primary care medical record and maternal interview for children aged 0-3 years: implications for research and clinical care', Ambulatory Pediatrics, 6(2), pp. 91-5.
152. Thabet, A. A. (2005) 'Validity of the arabic version of the general health questionnaire in the Gaza strip', PMJ - Palestinian Medical Journal, 1(1), pp. 33-36.
153. Thilagaratnam, S., Ding, Y. Y., Au Eong, K. G., Chiam, P. C., Chow, Y. L., Khoo, G., Lim, H. B., Lim, H. Y. L., Lim, W. S., Lim, W. Y., Peh, K. C., Phua, K. T., Sitoh, Y. Y., Tan, B. Y., Wong, S. F., Wong, W. P. and Yee, R. (2010) 'Health Promotion Board-Ministry of Health clinical practice guidelines: functional screening for older adults in the community', Singapore Med J, 50(6), pp. 508-21; quiz 522.
154. Tsai, A. C., Tomlinson, M., Dewing, S., le Roux, I. M., Harwood, J. M., Chopra, M. and Rotheram-Borus, M. J. (2014) 'Antenatal depression case finding by community health workers in South Africa: feasibility of a mobile phone application', Archives of Women's Mental Health, 17(5), pp. 423-31.
155. Van Cutsem, G., Ford, N., Hildebrand, K., Goemaere, E., Mathee, S., Abrahams, M., Coetzee, D. and Boulle, A. (2011) 'Correcting for mortality among patients lost to follow up on antiretroviral therapy in South Africa: a cohort analysis', PLoS ONE [Electronic Resource], 6(2), pp. e14684.
156. van der Linde, J., Swanepoel de, W., Glascoe, F. P., Louw, E. M. and Vinck, B. (2015) 'Developmental screening in South Africa: comparing the national developmental checklist to a standardized tool', African Health Sciences, 15(1), pp. 188-96.
157. Vogt, T. M., Aickin, M., Ahmed, F. and Schmidt, M. (2004) 'The Prevention Index: using technology to improve quality assessment', Health Services Research, 39(3), pp. 501-30.
158. Waheed, M. (1977) 'Management Information and Evaluation System (MIES) in IPP districts: a follow-up study', POPCEN News Letter, 3(4), pp. 1-5.
159. Were, M. C., Shen, C., Bwana, M., Emenyonu, N., Musinguzi, N., Nkuyahaga, F., Kembabazi, A. and Tierney, W. M. (2010) 'Creation and evaluation of EMR-based paper clinical summaries to support HIV-care in Uganda, Africa', International journal of medical informatics, 79(2), pp. 90-6.
160. Wunsch, G. (1983) 'Maternal and child health in the developing countries: problems of data collection', World Health Statistics Quarterly - Rapport Trimestriel de Statistiques Sanitaires Mondiales, 36(1), pp. 62-71.
161. Yellapurkar, M. V. (1984) 'Health Management Information System in Leprosy Control Programme', Indian Journal of Leprosy, 56(1), pp. 86-97.
162. Yut-Lin, W. and Othman, S. (2008) 'Early detection and prevention of domestic violence using the Women Abuse Screening Tool (WAST) in PHCclinics in Malaysia', Asia-Pacific Journal of Public Health, 20(2), pp. 102-16.
163. Zhong, Q. Y., Gelaye, B., Rondon, M. B., Sanchez, S. E., Simon, G. E., Henderson, D. C., Barrios, Y. V., Sanchez, P. M. and Williams, M. A. (2015) 'Using the Patient Health Questionnaire (PHQ-9) and the Edinburgh Postnatal Depression Scale (EPDS) to assess suicidal ideation among pregnant women in Lima, Peru', Archives of Women's Mental Health, 18(6), pp. 783-92.
