# Supplementary material for: Framework synthesis to inform the ideation and design of a paper‐based health information system (PHISICC)
Source: Int J Health Plann Manage. 2022 Apr 23;37(4):1953–72. doi: 10.1002/hpm.3487 (PMC9544999; doi:10.1002/hpm.3487)
Supplement: Supplementary file 4 — Supplementary Material [file HPM-37-1953-s002.docx]

**Supplementary file 4: Characteristics of Included Studies**

*Article title*: Research on Health Information Systems Focus on Technical Aspects Rather than on Decision-Making. A Framework Synthesis to Inform the Ideation of Paper-based Health Information Systems (PHISICC).

*Journal name*: IJHPM

*Authors information including author names, affiliation, and email address of the corresponding author*:

Meike-Kathrin Zuske^1, 2^, Christian Auer^1, 2^, Sandy Oliver^3,4^, John Eyers^5^, Xavier Bosch-Capblanch^1, 2^ ^*^

^1^ Swiss Tropical and Public Health Institute, Basel, Switzerland;

^2^ University of Basel, Basel, Switzerland

^3^ University College London, EPPI-Centre, Social Research Institute, London, United Kingdom

^4^ University of Johannesburg, Africa Centre for Evidence, Faculty of Humanities, Johannesburg, South Africa

^5^ Independent Consultant & Senior Research Fellow, 3ie, c/o LIDC, 20 Bloomsbury Square, London WC1A 2NS, United Kingdom

^*^ Correspondence: [x.bosch@unibas.ch](mailto:x.bosch@unibas.ch)

**Characteristics of Included Studies**

|  | **Reference** | **Country** | **Income level** | **Object** | **HIS** | **Participants** | **Health Subject** | **Setting** | **Type of study** |
| --- | --- | --- | --- | --- | --- | --- | --- | --- | --- |
| 1 | Abud-2015 | Brazil | UMIC | Data tool | Not defined | Combination | Child health | PHC | Cross Sectional |
| 2 | Al Baho-2003 | Kuwait | HIC | Data quality | Combination | Combination | General Practice | PHC | Cross Sectional |
| 3 | Alberti-2006 | Tunisia | UMIC | Data tool | Recording | Health care providers | Diabetes | PHC | Cross Sectional |
| 4 | Al-Hashimi-2014 | Bahrain | HIC | Data quality | Use of Health Information Technology | Health care providers | Infectious diseases | PHC | Cross Sectional |
| 5 | Alves-2009 | Brazil | UMIC | Data quality | Unclear | Health care providers | Child health | District | Cross Sectional |
| 6 | Amoakoh-Coleman-2015 | Ghana | LMIC | Data quality | Recording \| Reporting \| Assessment \| Transfer | Health care providers | Maternal health | Mixed | Cross Sectional |
| 7 | Azandegbe-2004 | Benin | LIC | Data tool | Assessment \| Recording | Health care providers | Maternal health | PHC | Cross Sectional |
| 8 | Barboza-2012 | Brazil | UMIC | Data tool | Recording | Health care providers | Health Information | PHC | Cross Sectional |
| 9 | Bogaerts-1995 | Rwanda | LIC | Data tool | Not defined | Health care providers | Cancer care or prevention | PHC | Cross Sectional |
| 10 | Broomhead-2011 | South Africa | UMIC | Data tool | Not defined | Manager | TB | PHC | Case-Control |
| 11 | Burke-2011 | Indonesia | LMIC | Data tool | Assessment | Combination | Maternal and child health | PHC | Mixed-method |
| 12 | da Costa-2009 | Brazil | UMIC | Data tool | Not defined | Patients | General Practice | PHC | Case-Control |
| 13 | Dalaba-2014 | Ghana | LMIC | Data tool | Outcome \| Recording \| Data audit | Health care providers | Maternal and child health | Unclear | Cross Sectional |
| 14 | DO-2009 | Tanzania | LIC | Data tool | Not defined | Patients | Child health | PHC | Cross Sectional |
| 15 | Doubova-2013 | Mexico | UMIC | Data tool | Technical audit | Health care providers | Hypertension | Unclear | Cross Sectional |
| 16 | Doubova-2014 | Mexico | UMIC | Data tool | Technical audit | Combination | Maternal and child health | Unclear | Mixed-method |
| 17 | Essen-1994 | Nicaragua | LMIC | Data tool | Not defined | Patients | Maternal and child health | PHC | Qualitative |
| 18 | Galvao-2008 | Brazil | UMIC | Combination | Several | Combination | Infectious diseases | Mixed | Mixed-method |
| 19 | Holanda-2012 | Brazil | UMIC | Data tool | Use of Health Information Technology | Health care providers | General Practice | PHC | Cross Sectional |
| 20 | Jimoh-2012 | Nigeria | LMIC | Data tool | Use of Health Information Technology | Health care providers | Maternal and child health | Unclear | Cross Sectional |
| 21 | Joubert-2013 | South Africa | UMIC | Data tool | Assessment \| Technical audit | Combination | Child health | PHC | Mixed-method |
| 22 | Kijsanayotin-2007 | Thailand | UMIC | Data tool | Use of Health Information Technology | Health care providers | Unclear | PHC | Cross Sectional |
| 23 | Kijsanayotin-2009 | Thailand | UMIC | Data tool | Use of Health Information Technology | Health care providers | Unclear | PHC | Cross Sectional |
| 24 | Kunimitsu-2009 | Solomon Islands | LMIC | Combination | | Combination | Malaria | Mixed | Cross Sectional |
| 25 | Leon-2015 | South Africa | UMIC | Data tool | Follow up | Patients | Hypertension | PHC | Qualitative |
| 26 | Lima-2010 | Brazil | UMIC | Data tool | Not defined | Health care providers | General Practice | PHC | Qualitative |
| 27 | Lungo-2008 | Tanzania | LIC | Combination | Use of Health Information Technology | Health care providers | Combination | Mixed | Mixed-method |
| 28 | Ly-2015 | Mali | LIC | Data tool | Recording | Health care providers | General Practice | Unclear | Cross Sectional |
| 29 | Mahmood-2010 | Pakistan | LMIC | Data quality | Recording \| Reporting \| Assessment | Health care providers | General Practice | PHC | Cross Sectional |
| 30 | Mahmood-2011 | Pakistan | LMIC | Data tool | Assessment \| Technical audit | Health care providers | Reproductive health | Unclear | Cross Sectional |
| 31 | Margalit-2006 | Israel | HIC | Data tool | Communication | Combination | General Practice | PHC | Cross Sectional |
| 32 | Mash-2007 | South Africa | UMIC | Data tool | Assessment \| Reporting | Health care providers | Diabetes | PHC | Cross Sectional |
| 33 | Mghamba-2004 | Tanzania | LIC | Combination | Data audit | Combination | Infectious diseases | Mixed | Mixed-method |
| 34 | Moimaz-2010 | Brazil | UMIC | Data quality | Reporting \| Data audit | Health care providers | Maternal health | Unclear | Cross Sectional |
| 35 | Odhiambo-Otieno-2005 | Kenya | LIC | Combination | Recording | Manager | Health Information | Unclear | Mixed-method |
| 36 | Ogwang-2009 | Uganda | LIC | Data tool | Assessment \| Recording \| Use of Health Information | Health care providers | Maternal health | Mixed | Mixed-method |
| 37 | Palombo-2014 | Brazil | UMIC | Data tool | Not defined | Combination | Child health | PHC | Cross Sectional |
| 38 | Parham-2010 | Zambia | LMIC | Data tool | Not defined | Health care providers | Cancer care or prevention | Unclear | Qualitative |
| 39 | Press-2009 | Israel | HIC | Data tool | Not defined | Health care providers | General Practice | PHC | Cohort |
| 40 | Queiroga-2011 | Brazil | UMIC | Combination | Combination | Health care providers | Family health | Unclear | Qualitative |
| 41 | Raeisi-2013 | Iran | UMIC | Combination | Combination | Combination | Health Information | District | Cross Sectional |
| 42 | Shachak-2009 | Israel | HIC | Data tool | Use of Health Information Technology | Health care providers | Unclear | PHC | Qualitative |
| 43 | Silva-2013 | Brazil | UMIC | Data tool |  | Health care providers | Family health | PHC | Qualitative |
| 44 | Sriha Belguith-2015 | Tunisia | UMIC | Data quality | Recording \| Reporting | Health care providers | General Practice | PHC | Cross Sectional |
| 45 | Tan-2009 | Singapore | HIC | Data tool | Use of Health Information Technology | Health care providers | General Practice | Unclear | Cross Sectional |
| 46 | Tarwa-2007 | South Africa | UMIC | Data tool | Not defined | Patients | Child health | PHC | Cross Sectional |
| 47 | Tierney-2007 | Kenya | LIC | Data tool | Not defined | Combination | HIV/Aids | PHC | Qualitative |
| 48 | Tseng-2010 | USA | HIC | Data tool | Knowledge \| Use of Health Information Technology | Health care providers | Health financing | Unclear | Cross Sectional |
| 49 | Vasconcellos-2008 | Brazil | UMIC | Data tool | Assessment \| Reporting | Health care providers | Combination | PHC | Cross Sectional |
| 50 | Wakgari-2015 | Ethiopia | LIC | Data tool | Assessment \| Action | Health care providers | Maternal and child health | Mixed | Cross Sectional |
