# Supplementary material for: Framework synthesis to inform the ideation and design of a paper‐based health information system (PHISICC)
Source: Int J Health Plann Manage. 2022 Apr 23;37(4):1953–72. doi: 10.1002/hpm.3487 (PMC9544999; doi:10.1002/hpm.3487)
Supplement: Supplementary file 5 — Supplementary Material [file HPM-37-1953-s005.docx]

**Supplementary file 5: Quality Assessment of Included Studies**

*Article title*: Research on Health Information Systems Focus on Technical Aspects Rather than on Decision-Making. A Framework Synthesis to Inform the Ideation of Paper-based Health Information Systems (PHISICC).

*Journal name*: IJHPM

*Authors information including author names, affiliation, and email address of the corresponding author*:

Meike-Kathrin Zuske^1, 2^, Christian Auer^1, 2^, Sandy Oliver^3,4^, John Eyers^5^, Xavier Bosch-Capblanch^1, 2^ ^*^

^1^ Swiss Tropical and Public Health Institute, Basel, Switzerland;

^2^ University of Basel, Basel, Switzerland

^3^ University College London, EPPI-Centre, Social Research Institute, London, United Kingdom

^4^ University of Johannesburg, Africa Centre for Evidence, Faculty of Humanities, Johannesburg, South Africa

^5^ Independent Consultant & Senior Research Fellow, 3ie, c/o LIDC, 20 Bloomsbury Square, London WC1A 2NS, United Kingdom

^*^ Correspondence: [x.bosch@unibas.ch](mailto:x.bosch@unibas.ch)

**Quality Assessment of Included Studies**

**(in red the 14 selected high-quality studies)**

| Reference | Aim | Context | Method | Sample | Outcome | Confounder | Analysis | Findings | Integration | Ethic | No other concerns | Score | Overall assessment |
| --- | --- | --- | --- | --- | --- | --- | --- | --- | --- | --- | --- | --- | --- |
| Palombo-2014 | Yes | Yes | Yes | Unclear | Yes | Yes | Yes | Yes | Yes | Yes | Yes | 10 | Minor concerns |
| Abud-2015 | Yes | Yes | Yes | Yes | Yes | Yes | Unclear | Yes | Yes | Yes | Yes | 10 | Minor concerns |
| dos Santos Ribeiro Silva-2013 | Yes | Yes | Yes | Unclear | Yes | No | Yes | Yes | Yes | Yes | Yes | 9 | Minor concerns |
| Margalit-2006 | Yes | Yes | Yes | No | Yes | Yes | Yes | Yes | Yes | No | Yes | 9 | Minor concerns |
| Alberti-2006 | Yes | Yes | Yes | Yes | Yes | Yes | Unclear | Yes | Yes | No | Yes | 9 | Minor concerns |
| Tseng-2010 | Yes | Yes | Yes | Unclear | Yes | Yes | Yes | Yes | Yes | No | Yes | 9 | Minor concerns |
| Doubova-2013 | Yes | Yes | Yes | Unclear | Yes | Yes | Yes | Yes | Unclear | Yes | Yes | 9 | Minor concerns |
| Wakgari-2015 | Yes | Yes | No | Yes | Yes | Yes | Yes | Yes | Yes | Yes | No | 9 | Minor concerns |
| Leon-2015 | Yes | Yes | Yes | Yes | Yes | No | Yes | Yes | Yes | Yes | No | 9 | Minor concerns |
| Ly-2015 | Yes | Yes | Yes | No | Yes | Yes | Yes | Yes | Yes | Yes | No | 9 | Minor concerns |
| Shachak-2009 | Yes | Yes | Yes | Yes | Yes | No | Yes | Yes | Yes | Yes | No | 9 | Minor concerns |
| Al-Hashimi-2014 | Yes | Yes | Yes | Yes | Yes | No | Yes | Yes | Yes | Yes | No | 9 | Minor concerns |
| Mahmood-2010 | Yes | Yes | Yes | Yes | Yes | No | Yes | Yes | Yes | Yes | No | 9 | Minor concerns |
| Amoakoh-Coleman-2015 | Yes | Yes | Yes | Yes | Yes | No | Yes | Yes | Yes | Yes | Yes | 10 | Minor concerns |
| Tarwa-2007 | Yes | Yes | Yes | No | Unclear | No | Yes | Yes | Yes | Yes | Yes | 8 | Minor to moderate concerns |
| Do-2009 | Yes | Yes | Yes | Unclear | Yes | Yes | No | Yes | Yes | Unclear | Yes | 8 | Minor to moderate concerns |
| Barboza-2012 | Unclear | Yes | Yes | Yes | Yes | No | No | Yes | Yes | Yes | No | 7 | Minor to moderate concerns |
| Essen-1994 | Yes | Yes | Yes | No | Unclear | No | No | Yes | Yes | No | Yes | 6 | Moderate concerns |
| Bogaerts-1995 | Yes | Yes | No | Yes | Yes | No | Unclear | Yes | Yes | No | No | 6 | Moderate concerns |
| Azandegbe-2004 | Yes | Yes | Yes | Yes | No | No | No | Yes | Yes | No | No | 6 | Moderate concerns |
| Kijsanayotin-2007 | Yes | Yes | Yes | Yes | Yes | No | Yes | Yes | No | No | Yes | 8 | Minor to moderate concerns |
| Mash-2007 | Yes | Yes | Yes | No | No | No | No | Yes | No | No | Yes | 5 | Moderate concerns |
| Vasconcellos-2008 | Unclear | Yes | Yes | No | Yes | No | Yes | Yes | No | Yes | No | 6 | Moderate concerns |
| Kijsanayotin-2009 | Yes | Yes | Yes | Yes | Yes | Yes | Yes | Unclear | No | Yes | No | 8 | Minor to moderate concerns |
| Tan-2009 | Yes | Yes | Yes | Yes | No | No | No | Yes | Yes | No | No | 6 | Moderate concerns |
| Ogwang-2009 | Yes | Yes | Yes | Yes | Yes | No | No | Unclear | Yes | Yes | No | 7 | Minor to moderate concerns |
| Broomhead-2011 | Yes | Yes | Yes | Unclear | No | No | Yes | Yes | No | No | Yes | 6 | Moderate concerns |
| da Costa-2009 | Yes | Yes | Yes | No | Yes | No | Yes | Yes | Unclear | Yes | No | 7 | Minor to moderate concerns |
| Moimaz-2010 | Yes | Yes | Yes | No | Unclear | No | Unclear | Yes | Unclear | Yes | No | 5 | Moderate concerns |
| Burke-2011 | Yes | Yes | Yes | No | Yes | No | Yes | Yes | No | No | No | 6 | Moderate concerns |
| Mahmood-2011 | Yes | Yes | Yes | Yes | Yes | No | No | Yes | Yes | Unclear | No | 7 | Minor to moderate concerns |
| Holanda-2012 | Yes | Yes | Yes | No | Yes | No | No | Yes | Yes | Unclear | Yes | 7 | Minor to moderate concerns |
| Jimoh-2012 | Yes | Yes | Yes | No | Yes | Yes | Unclear | Yes | Yes | No | Yes | 8 | Minor to moderate concerns |
| Joubert-2013 | Yes | Yes | Yes | Yes | Yes | No | No | Yes | No | Yes | Yes | 8 | Minor to moderate concerns |
| Doubova-2014 | Yes | Yes | Yes | No | Yes | No | No | Yes | Yes | Yes | No | 7 | Minor to moderate concerns |
| Dalaba-2014 | Yes | Yes | Yes | No | Yes | No | Unclear | Yes | Yes | Yes | Yes | 8 | Minor to moderate concerns |
| Raeisi-2013 | Yes | No | Yes | No | Yes | No | No | Yes | Yes | No | No | 5 | Moderate concerns |
| Mghamba-2004 | Yes | Yes | Yes | No | No | No | No | Yes | Yes | No | No | 5 | Moderate concerns |
| Al Baho-2003 | Yes | Yes | Yes | Yes | Yes | No | Yes | Yes | No | Yes | No | 8 | Minor to moderate concerns |
| Alves-2009 | Yes | Yes | No | Yes | Yes | No | Unclear | Yes | Yes | Yes | No | 7 | Minor to moderate concerns |
| Kunimitsu-2009 | Yes | Yes | Yes | Yes | Yes | No | Yes | Yes | No | No | Yes | 8 | Minor to moderate concerns |
| Sriha Belguith-2015 | Yes | Yes | Yes | No | Yes | No | Yes | Yes | Yes | No | No | 7 | Minor to moderate concerns |
| Lungo-2008 | Yes | Yes | No | Yes | Yes | No | No | Yes | No | No | No | 5 | Moderate concerns |
| Queiroga-2011 | Unclear | Unclear | Yes | No | No | Unclear | Yes | Unclear | No | Yes | No | 3 | Moderate to serious concerns |
| Odhiambo-Otieno-2005 | Yes | Yes | No | No | No | No | No | Yes | No | No | No | 3 | Moderate to serious concerns |
| Galvao-2008 | Yes | Yes | No | No | Yes | No | No | No | No | Yes | No | 4 | Moderate to serious concerns |
| Tierney-2007 | Yes | Yes | Yes | No | No | No | No | Yes | No | No | No | 4 | Moderate to serious concerns |
| Parham-2010 | Yes | Unclear | Yes | No | Unclear | No | No | No | No | No | Yes | 3 | Moderate to serious concerns |
| Press-2009 | Yes | Yes | Unclear | Unclear | No | No | Yes | No | No | No | Yes | 4 | Moderate to serious concerns |
| Lima-2010 | Yes | Yes | Yes | Unclear | No | Unclear | No | Unclear | No | Yes | No | 4 | Moderate to serious concerns |
